# Associations between Polygenic Risk for Psychiatric Disorders and Substance Involvement

**DOI:** 10.3389/fgene.2016.00149

**Published:** 2016-08-15

**Authors:** Caitlin E. Carey, Arpana Agrawal, Kathleen K. Bucholz, Sarah M. Hartz, Michael T. Lynskey, Elliot C. Nelson, Laura J. Bierut, Ryan Bogdan

**Affiliations:** ^1^Department of Psychological and Brain Sciences, Washington University in St. LouisSt. Louis, MO, USA; ^2^Department of Psychiatry, Washington University School of MedicineSt. Louis, MO, USA; ^3^Institute of Psychiatry, King’s College LondonLondon, UK

**Keywords:** substance, polygenic, comorbidity, schizophrenia, depression, cannabis, cocaine

## Abstract

Despite evidence of substantial comorbidity between psychiatric disorders and substance involvement, the extent to which common genetic factors contribute to their co-occurrence remains understudied. In the current study, we tested for associations between polygenic risk for psychiatric disorders and substance involvement (i.e., ranging from ever-use to severe dependence) among 2573 non-Hispanic European–American participants from the Study of Addiction: Genetics and Environment. Polygenic risk scores (PRS) for cross-disorder psychopathology (CROSS) were generated based on the Psychiatric Genomics Consortium’s Cross-Disorder meta-analysis and then tested for associations with a factor representing general liability to alcohol, cannabis, cocaine, nicotine, and opioid involvement (GENSUB). Follow-up analyses evaluated specific associations between each of the five psychiatric disorders which comprised CROSS—attention deficit hyperactivity disorder (ADHD), autism spectrum disorder (AUT), bipolar disorder (BIP), major depressive disorder (MDD), and schizophrenia (SCZ)—and involvement with each component substance included in GENSUB. CROSS PRS explained 1.10% of variance in GENSUB in our sample (*p* < 0.001). After correction for multiple testing in our follow-up analyses of polygenic risk for each individual disorder predicting involvement with each component substance, associations remained between: (A) MDD PRS and non-problem cannabis use, (B) MDD PRS and severe cocaine dependence, (C) SCZ PRS and non-problem cannabis use and severe cannabis dependence, and (D) SCZ PRS and severe cocaine dependence. These results suggest that shared covariance from common genetic variation contributes to psychiatric and substance involvement comorbidity.

## Introduction

Psychiatric disorders are genetically influenced complex traits, with heritability estimates ranging from 28% (generalized anxiety disorder) to 85% (bipolar disorder; Bienvenu et al., 2011). Accumulating evidence suggests that disruptions in common biological pathways may underpin multiple forms of psychopathology (Smoller, 2013). The Cross-Disorder Group of the Psychiatric Genomics Consortium (PGC^1^) recently identified common single nucleotide polymorphisms (SNPs) that jointly influence liability to five major mental disorders—attention deficit hyperactivity disorder (ADHD), autism spectrum disorder (AUT), bipolar disorder (BIP), major depressive disorder (MDD), and schizophrenia (SCZ)—and thus likely represent shared genetic etiology (Cross-Disorder Group of the Psychiatric Genomics Consortium, 2013; Lee et al., 2013). A more recent study performed by the Network and Pathway Analysis Subgroup of the PGC uncovered common genetic pathways underlying SCZ, BIP, and MDD (Network and Pathway Analysis Subgroup of Psychiatric Genomics Consortium, 2015), providing additional support for a biological contribution to shared liability to major psychiatric illnesses.

Though not included in PGC cross-disorder analyses, evidence suggests that substance use disorders are heritable (*h*^2^ = 40–70%; Kendler et al., 2012), frequently co-occur (Kendler et al., 2007), and are highly comorbid with other forms of psychopathology (Swendsen et al., 2010; Hasin and Kilcoyne, 2012). Such comorbidity is associated with increased severity and poorer outcomes for all disorders (Lehman et al., 1993), though it is unclear whether this relationship is causal (e.g., psychopathology leading to self-medication with substances, or substance use leading to psychopathology through dysregulation of neurotransmitter systems) or the result of overlapping risk factors (e.g., shared genetics and/or environment; Agrawal and Lynskey, 2014). Despite this, few studies have explored the role of shared genetic influence on comorbidity between substance use disorders and other psychiatric illnesses. Family studies remain equivocal about the co-transmission of substance use disorders and severe mental illness (e.g., schizophrenia and bipolar disorder: Kendler et al., 1993; Winokur et al., 1995); however, twin studies support the role of shared genetic liability for more common forms of psychopathology (Kendler et al., 2003). Collectively, these data hint at the possibility that genetic factors contributing to a range of psychopathologies also contribute to a general risk for substance involvement.

Though prior genetic studies of comorbidity have been necessarily limited in scope (e.g., to common disorders among related individuals), the development of polygenic risk scores (PRS; Purcell et al., 2009)—continuous indices of individual risk based on summary statistics from a genomewide association study (GWAS)—has allowed for examination of shared cross-trait genetic influence in unrelated and non-patient samples (e.g., Cross-Disorder Group of the Psychiatric Genomics Consortium, 2013; Krapohl et al., 2015). The aim of the current study was to examine general and specific genetic associations between psychiatric disorders and substance involvement using PRS derived from the PGC cross-disorder meta-analysis in a sample of 2573 non-Hispanic European–American participants ascertained for substance dependence in the Study of Addiction: Genetics and Environment (SAGE; Bierut et al., 2010). We first tested associations between cross-disorder polygenic risk (CROSS) and a general substance involvement factor (GENSUB). GENSUB was used due to evidence from twin studies that a large proportion of genetic liability is shared across substances (Kendler et al., 2003; Agrawal et al., 2012) and the use of a similar factor score in a prior GWAS (Wetherill et al., 2015). Because substance use disorders are highly comorbid with other forms of psychopathology (Swendsen et al., 2010; Hasin and Kilcoyne, 2012), with evidence of shared genetic and environmental risk factors (Kendler et al., 2003; Agrawal et al., 2012), we hypothesized that increased cross-disorder polygenic risk would be associated with greater general substance involvement. Next, we tested individual associations between PRS for each of the five psychiatric disorders included in the cross-disorder meta-analysis (ADHD, AUT, BIP, MDD, and SCZ) and involvement with the five substances assessed in SAGE (alcohol, cannabis, cocaine, opioids, and nicotine). Finally, we tested whether these associations were substance-specific or best explained by association with the general substance involvement factor.

## Materials and Methods

### Sample

Non-Hispanic European–American adults who completed the Study of Addiction: Genetics and Environment (SAGE; Bierut et al., 2010) were included in analyses (*N* = 2573; see **Table 1** for demographic information; see Supplementary Materials and Methods for details regarding ancestry determination). Alcohol dependent (*n* = 1160; required 12-month clustering of DSM-IV symptoms) and control (*n* = 1413) participants were recruited from three large, complementary datasets ascertained for alcohol (Collaborative Study of the Genetics of Alcoholism; Reich et al., 1998; Foroud et al., 2000), nicotine (Collaborative Study of the Genetics of Nicotine Dependence; Bierut et al., 2007; Saccone et al., 2007), and cocaine (Family Study of Cocaine Dependence; Bierut et al., 2008) dependence. Alcohol dependent cases often met criteria for a variety of other substance use disorders. Controls did not meet criteria for alcohol dependence or for cocaine, cannabis, and opioid dependence (nicotine dependence was allowed) but may have used these substances and endorsed some symptoms at non-diagnostic levels. The Institutional Review Board at each contributing institution (i.e., Henry Ford Health Sciences Center, Howard University, Indiana University, SUNY Health Sciences Center at Brooklyn, University of California—San Diego, University of Connecticut Health Center, University of Iowa, and Washington University in St. Louis) reviewed and approved the protocols for genetic studies under which all participants were recruited. All participants gave written informed consent in accordance with the Declaration of Helsinki.

**Table 1:** Sample demographics.

Demographics	
Female	56.2%
Age	38.67 (9.76)
Study of Origin (*n* Participants)	
COGA	927
FSCD	557
COGEND	1089

*COGA, Collaborative Study of the Genetics of Alcoholism; FSCD, Family Study of Cocaine Dependence; COGEND, Collaborative Study of the Genetics of Nicotine Dependence*.

### Measures

Participants completed a version of the Semi-Structured Assessment for the Genetics of Alcoholism (Bucholz et al., 1994), wherein lifetime DSM-IV substance dependence symptoms were assessed for alcohol, cannabis, cocaine, nicotine, and opioids. As genes influencing liability to substance use initiation may only partially overlap with genes influencing progression to various levels of dependence (Heath et al., 2002), categorical measures (five levels) for each substance were created to represent differential levels of involvement: (A) no lifetime (cannabis, cocaine, opioids) or non-regular (alcohol, nicotine) use, (B) non-problem use (i.e., use without endorsement of any dependence symptoms), (C) mild problems (i.e., 1–2 dependence symptoms), (D) moderate dependence (i.e., 3–5 dependence symptoms), and (E) severe dependence (i.e., 6–7 dependence symptoms; see **Table 2** for distributions of participants across involvement levels). The lowest level of involvement was used as the reference group, though all groups were compared to one another (see Statistical Analyses). For cannabis, cocaine, and opioids, the reference group included individuals with no lifetime history of using the substance; for alcohol, those who had never drank at least once per month for 6 months or longer were considered to be minimally/not exposed, while for nicotine, this threshold was set at having smoked less than 100 cigarettes. The vast majority of individuals (82.9%) reported using multiple substances during their lifetime, with 17.0% reporting use of all substances assessed. Only 5.7% of the sample belonged to all substance-specific reference groups, reflecting no lifetime use of cannabis, cocaine, and opioids, and no regular use of alcohol and nicotine. Lifetime histories of problematic substance use also co-occurred, with 62.1% of the sample reporting at least one dependence symptom for two or more substances. Finally, in addition to alcohol dependence (46.9%), 17.4, 18.6, 50.9, and 6.9% of the sample endorsed 3 or more dependence criteria (unclustered) for cannabis, cocaine, nicotine, and opioid dependence, respectively (**Table 2**). A measure of general substance involvement (GENSUB) was generated by performing a confirmatory factor analysis (CFA) on the individual substance involvement measures in Mplus (v.7.11; Muthén and Muthén, 2015) and standardizing the resulting factor score (Supplementary Figure S2).

**Table 2:** Substance involvement distributions.

Involvement Group	Alcohol	Nicotine	Cannabis	Cocaine	Opioids
No/Non-Regular Use^a^	258	595	659	1591	2043
Use, 0 Symptoms	517	159	1153	416	305
Use, 1–2 Symptoms	591	499	312	83	45
Use, 3–5 Symptoms^b^	648	1120	278	131	52
Use, 6–7 Symptoms	559	180	168	348	124

*^a^For illicit drugs (i.e., cannabis, cocaine, and opioids), the lowest level of involvement included individuals who had never used that particular substance; for licit drugs (i.e., alcohol and nicotine), individuals who had also never used the substance regularly (i.e., drinking at least once per month for 6 months, or smoking 100+ cigarettes in one’s lifetime) were also included in the lowest involvement category. ^b^Symptoms were not required to cluster within a 12-month period*.

### Genotyping Methods and Quality Control

DNA was extracted from blood samples, and cell lines were developed as an additional DNA source. Samples were genotyped using lllumina Human1Mv1_CBeadChip at the Johns Hopkins Center for Inherited Disease Research (CIDR). Extensive and rigorous data cleaning was employed (Laurie et al., 2010), resulting in quality-controlled genotypic data for 948,658 SNPs (Bierut et al., 2010).

### Polygenic Risk Scores

Polygenic risk scores were derived from the results of the PGC cross-disorder meta-analysis (CROSS; Cross-Disorder Group of the Psychiatric Genomics Consortium, 2013) of 5 psychiatric disorders (ADHD, AUT, BIP, MDD, SCZ). PRS were constructed for the following *p*-value thresholds based on the full GWAS summary statistics: 0.0001, 0.001, 0.01, 0.05, 0.1, 0.2, 0.3, 0.4, 0.5, and 1.0; these thresholds were selected to be consistent with the PRS analyses conducted in the PGC cross-disorder paper (Cross-Disorder Group of the Psychiatric Genomics Consortium, 2013). The PRS generation pipeline was coded in Python (v.2.7.6) using the Numerical Python (“NumPy,” v.1.7.1), StatsModels (v.0.5.0), and Python Data Analysis (“pandas,” v.0.12.0) libraries. Single Nucleotide Polymorphisms (SNPs) were required to have a MAF > 0.02, call rates > 0.98, and HWE *p*-values >10^-6^ to be included in analyses. SNPs within the MHC region (chr6: 25000000:35000000) were excluded due to their complex patterns of linkage disequilibrium. All remaining SNPs were then pruned using *p*-value-informed clumping (i.e., grouping linked SNPs; *R*^2^ = 0.10, 500 kb window), leaving 101,202 SNPs in SAGE for analysis. For each *p*-value threshold, the cross-disorder log odds-ratio for each component SNP was multiplied by the number of reference alleles for that SNP. These product terms were summed and divided by the total number of contributing SNPs, thus producing a single metric for each participant representing cross-disorder genetic vulnerability. These analyses were completed using the –score method in Plink (v.1.9; Chang et al., 2015). Individual disorder risk scores for the five psychiatric disorders (ADHD, AUT, BIP, MDD, SCZ) included in the cross-disorder meta-analysis were then generated in the same manner. Subsequent to the cross-disorder meta-analysis, a much larger second-generation GWAS of schizophrenia (SCZ2; Schizophrenia Working Group of the Psychiatric Genomics Consortium, 2014) was released, and analyses were therefore repeated for schizophrenia using SCZ2 PRS. Distributions of all PRS generated are presented in Supplementary Figure S1.

### Statistical Analyses

Associations between each thresholded CROSS PRS and GENSUB were tested using ordinary least squares regression. Multinomial logistic regression was then used to test associations across each level of involvement (i.e., no/non-regular use, non-problem use, mild problems, moderate dependence, and severe dependence) for specific substances and individual disorder PRS. Due to the large number of non-independent tests performed, an empirical significance threshold for α = 0.05 was determined using 10,000 label-swapping permutations (see Supplementary Materials and Methods for details). The lowest level of involvement was used as the reference group; thus, resulting odds-ratios (ORs) reflect increases or decreases in association for each level of substance involvement relative to the lowest level (i.e., no/non-regular use). Wald chi-square tests (for 1° of freedom) were used to examine whether the magnitude of these resulting ORs could be equated to each other and thus establish whether differences in PRS existed across involvement levels (e.g., comparison of the OR between no use and use with no problems vs. the OR between no use and use with 1–2 symptoms). To determine whether specific disorder-substance associations were driven by GENSUB, significant analyses were repeated with GENSUB as a covariate. Covariates across all analyses included sex, age quartiles, three ancestrally informative principal components, and study of origin.

## Results

### GENSUB Confirmatory Factor Analysis

The confirmatory one-factor model fit the data reasonably well in our sample (comparative fit index = 0.992; root mean square error of approximation = 0.106), supporting our proposed unidimensional conceptualization of alcohol, cannabis, cocaine, nicotine, and opioid involvement. Factor loadings were generally comparable across substances, though the loading for nicotine was somewhat lower (Supplementary Figure S2).

### CROSS PRS and GENSUB

**FIGURE 1:**
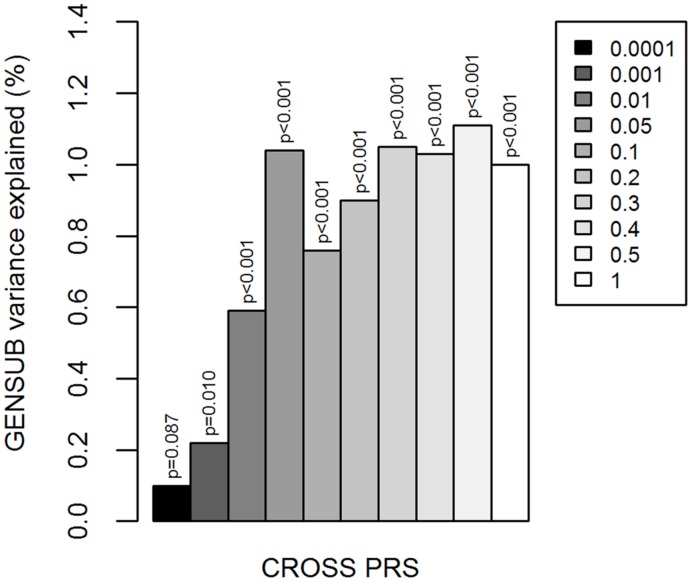
**Cross-disorder polygenic risk scores and general substance involvement liability.** *Y*-axis is the percent of variation in GENSUB explained by CROSS PRS. Shades of gray in legend indicate the *p*-value threshold (i.e., *p* < 0.0001, 0.001, 0.01, 0.05, 0.1, 0.2, 0.3, 0.4, 0.5, or 1.0) at which the risk score was calculated based on the results of the original cross-disorder meta-analysis. CROSS, cross-psychiatric-disorder. GENSUB, general substance involvement liability. PRS, polygenic risk score.

CROSS PRS were associated with increasing GENSUB factor scores (significant at 9 of 10 *p*-value thresholds; most significant at *p* < 0.5: β^∗^ = 0.110, *R*^2^ = 0.011, *p* < 0.001; **Figure 1**; Supplementary Table S1), indicating a positive relationship between genetic liability to multiple psychiatric disorders and general substance involvement.

### Specific Disorder-Substance Associations

**FIGURE 2:**
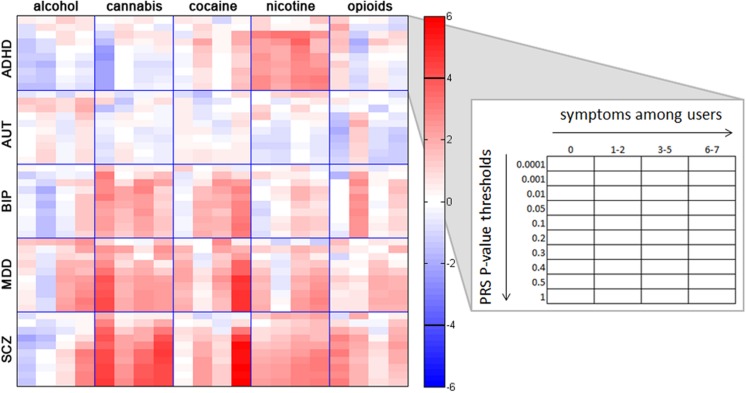
**Associations between individual substance involvement and polygenic risk scores (PRS) for five major psychiatric disorders.** Within each grid space, *p*-thresholds at which PRS were calculated (i.e., *p* < 0.0001, 0.001, 0.01, 0.05, 0.1, 0.2, 0.3, 0.4, 0.5, and 1.0) are represented vertically in ascending order. Levels of involvement (i.e., no/non-regular use, use without endorsement of any dependence symptoms, 1–2 dependence symptoms, 3–5 dependence symptoms, and 6–7 dependence symptoms) are represented horizontally in ascending order. Colors represent *z*-scores for each association test, with no lifetime or nonregular use as the reference group. For example, the red colors in the crosstab between SCZ and cannabis indicate a high correlation between genetic risk for SCZ and cannabis involvement. The black horizontal bars in the color bar indicate the approximate *z*-score cutoff for significance post-correction for multiple comparisons (*z* = ± 3.911). Post-hoc Wald tests comparing all levels of substance involvement with one another are reported in Supplementary Tables S2–S7. ADHD, attention deficit hyperactivity disorder; AUT, autism; BIP, bipolar disorder; MDD, major depressive disorder; SCZ, schizophrenia.

Analyses of individual disorder PRS and specific substance involvement revealed several noteworthy associations (see **Figure 2**; Supplementary Tables S2–S7 for all associations), some of which remained significant when controlling for general substance involvement (i.e., GENSUB; Supplementary Tables S2–S7). We report all nominally significant associations below and note which survived permutation-based correction for multiple comparisons within the text and in Supplementary Tables S2–S7.

#### Attention Deficit Hyperactivity Disorder

ADHD PRS were negatively associated with non-problem cannabis use, and were positively associated with all levels of nicotine use (Supplementary Table S2). No associations remained significant after correction for multiple testing. ***Cannabis:*** Compared with nonusers, non-problem cannabis users (i.e., users with 0 symptoms) had lower ADHD PRS. ***Nicotine:*** Users at all levels of nicotine involvement (i.e., non-problem use, mild problems, and moderate and severe dependence) had elevated ADHD PRS relative to nonusers. Despite associations across increasing levels of nicotine involvement, the magnitude of these associations did not statistically differ from each other.

#### Autism Spectrum Disorder

AUT PRS were not consistently associated with involvement with any of the substances tested (Supplementary Table S3).

#### Bipolar Disorder

BIP PRS were associated with increasing problematic alcohol involvement, severe cocaine dependence, and specific levels of cannabis and opioid involvement (Supplementary Table S4). None of these associations survived correction for multiple comparisons. ***Alcohol:*** There was evidence for a dose-dependent relationship between BIP PRS and increasing number of alcohol dependence symptoms among regular drinkers with at least one symptom of dependence. ***Cannabis:*** Compared with those who had never used cannabis, BIP PRS were higher and of a similar magnitude in individuals with non-problem cannabis use and moderate dependence. ***Cocaine:*** Relative to non-users and non-problem users, those with severe cocaine dependence exhibited higher BIP PRS. ***Opioids:*** Elevated BIP PRS were associated only with mild problems (i.e., 1–2 dependence symptoms).

#### Major Depressive Disorder

MDD PRS were associated with increased alcohol, cocaine, and nicotine involvement, as well as with multiple levels of cannabis involvement (Supplementary Table S5). Associations with non-problem cannabis use and severe cocaine dependence remained significant following correction for multiple tests. ***Alcohol:*** MDD PRS were associated with moderate and severe alcohol dependence relative to no/non-regular use and lesser levels of involvement. ***Cannabis:*** Higher MDD PRS differentiated cannabis users with 0, 3–5, and 6–7 dependence symptoms from non-users. ***Cocaine:*** Compared with non-users and non-problem users, those with severe cocaine dependence had higher MDD PRS. ***Nicotine:*** MDD PRS were associated with moderate and severe nicotine dependence relative to non-regular use and lower levels of involvement.

#### Schizophrenia

SCZ PRS were associated with elevated alcohol, cannabis, and cocaine involvement, along with nicotine use and non-problem opioid use (Supplementary Table S6). Relationships with non-problem cannabis use, severe cannabis dependence, and severe cocaine dependence survived multiple comparison correction. Associations with SCZ2 PRS were comparable and are described in the Supplementary Results (see also Supplementary Table S7; see Supplementary Figure S3 for a visual comparison to SCZ results). ***Alcohol:*** Elevated SCZ PRS were associated with severe alcohol dependence relative to all other categories of involvement (i.e., non-regular use, non-problem use, and use with fewer dependence symptoms). ***Cannabis:*** Elevated SCZ PRS were associated with cannabis use at all levels of involvement relative to non-use and differentiated those with severe dependence from non-problem users and users with mild problems. ***Cocaine:*** Higher SCZ PRS differentiated cocaine users with 1–2 and 6–7 dependence symptoms from non-users, and those with 6–7 symptoms from those with fewer (i.e., 0 and 3–5) symptoms. ***Nicotine:*** Elevated SCZ PRS were associated with nicotine use at all levels of involvement. ***Opioids:*** Higher SCZ PRS were associated with non-problem opioid use.

#### Associations Not Attributable to GENSUB

Controlling for GENSUB revealed that the majority of nominally significant substance-disorder relationships were driven by associations between PRS and general substance involvement liability, though a few substance-disorder pairings appear to be specific: ***ADHD PRS*** with all levels of nicotine involvement and non-problem cannabis use; ***BIP PRS*** with non-problem cannabis use; ***MDD PRS*** with alcohol dependence relative to use with mild problems, as well as severe cocaine dependence relative to never-use and non-problem use, and severe cannabis dependence relative to never-use (though, contrary to expectations, this residual association was negative); and ***SCZ PRS*** with non-problem cannabis use as well as severe cocaine dependence relative to non-use and use with 0 and 3–5 dependence symptoms.

## Discussion

The substantial comorbidity between psychiatric and substance use disorders is unequivocal (e.g., Swendsen et al., 2010; Hasin and Kilcoyne, 2012), but sources contributing to this covariation remain less well articulated. Complementing prior observations from latent genetic (e.g., Kendler et al., 2003) and candidate gene (e.g., Yoshimasu et al., 2015) studies, we report that PRS derived from the PGC cross-disorder meta-analysis (CROSS; Cross-Disorder Group of the Psychiatric Genomics Consortium, 2013) explained roughly 1% of the variance in general substance involvement (GENSUB) in our target sample enriched for substance use (SAGE; Bierut et al., 2010). This effect size is consistent with previously published cross-trait PRS analyses (e.g., Cross-Disorder Group of the Psychiatric Genomics Consortium, 2013; Krapohl et al., 2015), and, though not large enough to be informative on an individual level, nonetheless provides support for the hypothesized role of shared genetics in the lifetime co-occurrence of psychiatric and substance use disorders. PRS for individual psychiatric diagnoses (i.e., ADHD, BIP, MDD, and SCZ, but not AUT) were also significantly associated with specific substance involvement (i.e., alcohol, cannabis, cocaine, nicotine, and opioids), and are discussed in detail below. As in **Results**, all nominally significant substance-specific individual psychiatric diagnosis PRS associations are discussed to provide an overview of observed associations. Only the following specific PRS and substance use associations survived permutation-based correction for multiple comparisons: (A) MDD PRS with non-problem cannabis use, (B) MDD PRS with severe cocaine dependence, (C) SCZ PRS with non-problem cannabis use and severe cannabis dependence, and (D) SCZ PRS with severe cocaine dependence.

### Specific Disorder-Substance Associations

Associations between PRS and individual substances were only partially attributable to GENSUB, indicating specificity of certain relationships (e.g., ADHD PRS and nicotine involvement). This is significant, considering that twin studies implicate GENSUB as the primary source of genetic variance in individual substance use disorders (Kendler et al., 2003, 2007, 2012). Our factor loadings support this high degree of cohesiveness, with the possible exception of nicotine, also consistent with a prior twin study (Kendler et al., 2007). Notably, unlike prior research which has heavily relied on binary measures of substance use or dependence, we capitalized on the range of substance involvement present in our target sample due to the ascertainment strategy (**Table 2**), including non-problem use (i.e., use with no dependence symptoms) as well as multiple levels of problematic use (i.e., 1–2 vs. 3–5 vs. 6–7 dependence symptoms). Not only did this coding allow us to differentiate between early/casual and later/maladaptive levels of substance involvement, but it also allowed us to test whether PRS were associated with specific levels of substance involvement (i.e., to compare across groups). Despite factorial architecture suggesting only modest residual variance, several PRS remained associated with individual substances, particularly at non-problem or severe levels of involvement, even after accounting for this shared liability. This overall finding suggests that despite genetic susceptibility across substances being largely shared, variation at the extremes of the phenotype may be less well captured by measures such as GENSUB.

#### Attention Deficit Hyperactivity Disorder

ADHD PRS were associated with nicotine and cannabis involvement, even after controlling for GENSUB. These findings are markedly consistent with an expansive epidemiological and clinical literature documenting higher rates of cigarette smoking in individuals with ADHD, even after accounting for comorbid conduct problems (e.g., Elkins et al., 2007; Chang et al., 2012). Consistent with prior studies showing risk effects of ADHD on both smoking initiation and dependence (e.g., Elkins et al., 2007; Sibley et al., 2014), ADHD PRS related to all levels of nicotine involvement. However, these associations were all of a similar magnitude, allowing us to conclude that the relationship was not dose-dependent with respect to severity of involvement. In contrast, we noted a negative relationship between ADHD PRS and non-problem cannabis use. The evidence supporting the role of ADHD in the use of cannabis is stronger than support for its role in the onset of cannabis use disorders (e.g., Elkins et al., 2007; Lee et al., 2011). However, we are not aware of any studies that have examined non-problem use specifically. It is possible that individuals at high genetic liability to ADHD are less likely to engage in non-problem use and, consistent with the literature, not at a particularly elevated risk of progression to problem use.

#### Autism Spectrum Disorder

The lack of association between AUT and substance involvement was unsurprising given a mixed literature linking autism spectrum disorders to relatively reduced (e.g., Abdallah et al., 2011) or elevated (De Alwis et al., 2014) risk of substance involvement.

#### Bipolar Disorder

Our findings of positive associations between BIP PRS and multiple levels of alcohol, cocaine, cannabis, and opioid involvement are consistent with observations of markedly elevated rates of substance use and use disorders in individuals with BIP (Compton et al., 2007), as well as with prior studies indicating a genetic origin of this comorbidity (Biederman et al., 2000; Johnson et al., 2009). Notably, when controlling for GENSUB, only a positive association between BIP PRS and non-problem cannabis use remained significant. This overall association with general, rather than specific, substance involvement liability may be reflective of similar cognitive mechanisms (e.g., impulsivity, emotion dysregulation, sensation-seeking) that are thought to broadly underlie both BIP and substance use disorders (Swann, 2010).

#### Major Depressive Disorder

Elevated polygenic liability to MDD in our sample was associated with increasing problematic use of alcohol, cocaine, nicotine, and cannabis, in-line with prior twin studies suggesting MDD shares genetic liability with alcohol (Prescott et al., 2000), nicotine (Edwards et al., 2011), and cannabis use (Lynskey et al., 2004). Associations between MDD PRS and alcohol and cocaine dependence remained even when controlling for GENSUB. Prior genomewide studies of MDD with comorbid alcohol and cocaine dependence have uncovered significant or near-significant overlapping regions/variants contributing to MDD alone and to MDD with a comorbid SUD, as well as some regions/variants contributing to a combined MDD and SUD phenotype only (Yang et al., 2011; Edwards et al., 2012). Taken together, these parallel lines of evidence suggest that relationships between MDD and alcohol and cocaine dependence are substance-specific.

#### Schizophrenia

SCZ PRS were associated with involvement across all substances tested, but only associations with non-problem cannabis use and severe cocaine dependence persisted upon inclusion of GENSUB. Notably, both substances have been previously implicated in the etiology of psychotic illness. Cocaine use is common among individuals with SCZ (Shaner et al., 1995), and several neurobiological models have implicated shared disruptions in dopaminergic signaling as a common etiological explanation for this comorbidity (e.g., Volkow, 2009). Adolescent exposure to cannabis has long been posited as either a directly causal (Arseneault et al., 2004) or moderating (Henquet et al., 2008) factor in the etiology of psychosis, but recent research has suggested that associations may be due in part to shared genetic factors (Power et al., 2014; Pagliaccio et al., 2015). For example, complementing our current results, a prior study reported a significant association between SCZ PRS and lifetime and frequency of cannabis use (Power et al., 2014). Taken together, these results suggest a specific genetic etiological link between schizophrenia, cannabis use, and cocaine dependence.

### Limitations

Some limitations of our study are noteworthy. First, comorbidities in the cross-disorder meta-analysis from which the PRS were derived, as well as in the target SAGE sample, may be subject to certain unmeasured confounds. For example, the PGC did not examine the extent of cocaine (or other substance) use in their sample population (Cross-Disorder Group of the Psychiatric Genomics Consortium, 2013), so our associations of SCZ PRS with severe cocaine dependence may be an artifact of increased cocaine use by people with schizophrenia (Serper et al., 1995), or of cocaine-induced psychosis resulting in a diagnosis of schizophrenia (Brady et al., 1991). Conversely, though severe psychopathology (i.e., AUT, BIP, SCZ) is likely to be uncommon in SAGE and unlikely to influence associations, more common psychopathologies (i.e., ADHD, MDD) were likely present. Therefore, associations with ADHD or MDD PRS may have been mediated by the actual expression of the disorder among SAGE participants (e.g., people with higher MDD PRS in SAGE develop MDD, which in turn is associated with substance use disorders). Though data on ADHD diagnosis in SAGE were unavailable, repeating MDD PRS analyses while controlling for DSM-IV MDD diagnosis did not alter results, indicating that associations between PRS and substance use outcomes were not directly related to disorder expression (Supplementary Table S8). Longitudinal studies of well-characterized populations, as well as an increased emphasis on the study of substance use disorders in consortia such as the PGC, will be critical to further address these questions of temporality and comorbidity.

Second, SAGE was ascertained for liability to substance dependence, specifically to alcohol, nicotine, and cocaine; the generalizability of these findings to the general population is thus unclear. Additionally, the factor structure of GENSUB might be somewhat sample-specific, and residual associations with involvement with specific substances (i.e., non-problem cannabis use) may have been artifacts of sample ascertainment. However, this ascertainment strategy allowed us to study the full range of substance involvement—from never-use to severe dependence—across both licit (i.e., alcohol, nicotine) and illicit (i.e., cannabis, cocaine, opioids) drugs, which would not have been possible in a population-based sample of comparable size. Nonetheless, it is important to replicate these findings in other samples.

Third, though multiple nominally significant relationships between genetic risk for individual psychiatric disorders and involvement with specific substances emerged, few survived correction for the large number of statistical tests performed. These results thus may represent spurious associations and should be interpreted with caution. However, given the consistency of certain associations (e.g., ADHD and nicotine use) with prior genetic (e.g., Chang et al., 2012) and epidemiological (e.g., Elkins et al., 2007; Lee et al., 2011) literature, they should not be dismissed outright. Notably, the only disorders with significant post-correction associations—MDD and SCZ—were those with the largest numbers of cases in the PGC cross-disorder meta-analysis (*N*_MDD_ = 9227, *N*_SCZ_ = 9379). Nominal associations may thus strengthen with larger discovery samples, which may provide more precise PRS estimates, as well as larger target samples. In support of this interpretation, repetition of schizophrenia PRS analyses with scores derived from the much larger second-generation PGC GWAS (*N*_SCZ2_ = 36,989) yielded associations that were generally stronger than those from the first-generation analyses (Supplementary Tables S6 and S7; see Supplementary Figure S3 for comparison).

Fourth, while our PRS approach yielded evidence that shared common genetic architecture contributes to comorbidity between psychopathology and substance involvement, it does not provide insight into specific biological (e.g., reward-related neural responsiveness, epigenetically medicated changes in gene expression), psychological (e.g., anhedonia, impulsivity), and/or experiential (e.g., early life stress, peer group) mechanisms through which this risk is manifest (e.g., Olfson et al., 2014; Peña-Oliver et al., 2016; Ron and Barak, 2016). Compelling evidence suggests that psychopathology and substance involvement share overlapping neural systems (e.g., Buckholtz and Meyer-Lindenberg, 2012), molecular pathways (e.g., Ng et al., 2013), and environmental exposures (e.g., Kristjansson et al., 2016). Future PRS research probing biological systems and psychological traits common to both psychiatric and substance use disorders (e.g., Lancaster et al., 2016; Peña-Oliver et al., 2016) and incorporating environmental/experiential measures (e.g., French et al., 2015), alongside genomewide efforts to partition heritability into specific pathways and functional categories (e.g., Lee et al., 2012; Finucane et al., 2015), will further our understanding of the mechanisms underlying this comorbidity (Bogdan et al., 2016).

## Conclusion

Our study provides some of the first evidence that common polygenic liability to major psychiatric disorders is related to use and misuse of licit and illicit substances, providing new insights into the etiology of this well documented comorbidity. Future efforts might attempt to determine which specific biological pathways and networks underlie this shared genetic variance, or prospectively evaluate the predictive power of such PRS: for instance, whether polygenic risk for SCZ predicts onset, severity, and prognosis of illness in youth who experiment with cannabis and other drugs. Additionally, the inclusion of a substance use disorders workgroup in the second iteration of the PGC^2^ promises to provide substantially larger sample sizes in which the current work may be replicated and extended.

## Author Contributions

CC, AA, and RB were responsible for the conception and design of the study. CC performed all analyses. CC, AA, and RB drafted the manuscript. LB collected the SAGE dataset and, along with SH, EN, KB, and ML, provided expertise on analyses. All authors critically reviewed content and provided feedback.

## Conflict of Interest Statement

LB is listed as an inventor on Issued U.S. Patent 8,080,371, “Markers for Addiction” covering the use of certain SNPs in determining the diagnosis, prognosis, and treatment of addiction. All the other authors declare that the research was conducted in the absence of any commercial or financial relationships that could be construed as a potential conflict of interest. The reviewer RB and handling Editor declared their shared affiliation, and the handling Editor states that the process nevertheless met the standards of a fair and objective review.

## Footnotes

1 http://www.med.unc.edu/pgc

2 http://pgc-sud.med.wustl.edu/
