# Supplementary material for: Associations between Polygenic Risk for Psychiatric Disorders and Substance Involvement
Source: Front Genet. 2016 Aug 15;7:149. doi: 10.3389/fgene.2016.00149 (PMC4983546; doi:10.3389/fgene.2016.00149)
Supplement: Supplementary file 1 [file Data_Sheet_1.DOCX]

**SUPPLEMENTARY MATERIAL**

**Associations between Polygenic Risk for Psychiatric Disorders and Substance Involvement**

Caitlin E. Carey^*^, Arpana Agrawal, Kathleen K. Bucholz, Sarah M. Hartz, Michael T. Lynskey, Elliot C. Nelson, Laura J. Bierut, Ryan Bogdan^*^

***Correspondence:**

Caitlin E. Carey (caitlin.carey@wustl.edu)

Ryan Bogdan (rbogdan@wustl.edu)

**Contents**

| Supplementary Materials and Methods | 2 |
| --- | --- |
| Supplementary Results | 2 |
| Supplementary References | 2 |
| Table S1. CROSS PRS and GENSUB | 4 |
| Table S2. Individual Substance Associations with ADHD PRS | 5 |
| Table S3. Individual Substance Associations with AUT PRS | 7 |
| Table S4. Individual Substance Associations with BIP PRS | 9 |
| Table S5. Individual Substance Associations with MDD PRS | 11 |
| Table S6. Individual Substance Associations with SCZ PRS | 13 |
| Table S7. Individual Substance Associations with SCZ2 PRS | 15 |
| Table S8. Individual Substance Associations with MDD PRS, Controlling for MDD Diagnosis | 17 |
| Figure S1. PRS Distributions at P<0.3 | 19 |
| Figure S2. Factor Loadings for the Confirmatory One-Factor Model of GENSUB | 20 |
| Figure S3. Comparison of CROSS SCZ to SCZ2 PRS Results | 21 |

**1. SUPPLEMENTARY MATERIALS AND METHODS**

**1.1 Ancestry Determination**

To verify self-reported race and ethnicity and account for differences in ancestral background, we generated ancestrally informative principal components using EIGENSTRAT (v.5.0.1).[^1^](#_ENREF_1) Visual inspection of the scree plot of the eigenvalues of the top 10 components revealed a point of inflection at the third principal component, indicating that the top 3 principal components account for divergent ancestral groups within the population. Twenty-one participants were identified as ancestral outliers, as they were greater than 6 standard deviations from the mean on these top 3 components, and were excluded from analyses. Thus, a relatively ancestrally homogeneous final sample of 2,573 self-reported non-Hispanic European-American participants (**Table 1**),[^2^](#_ENREF_2) validated using principal component analyses, was analyzed using the top 3 principal components as covariates to further account for divergent ancestry within the sample.

**1.2 Calculation of Empirical Significance Threshold**

A large number of tests were carried out to assess the relationships between specific disorder PRS and substance involvement (i.e., 5 disorders * 10 thresholds * 5 substances * 4 levels of involvement = 1000 tests total). However, these tests were not truly independent, as the PRS thresholds were nested (i.e., less stringent PRS thresholds included all SNPs from the more stringent thresholds), and each participant could only belong to one of 5 substance involvement levels (i.e., including the reference group). Thus, to determine the appropriate threshold for significance, 10,000 label-swapping permutations were performed, whereby each participant’s PRS and ancestrally informative principal components were kept intact, while their substance involvement scores and additional covariates were swapped. For each swapped dataset, all association tests were run, and the largest absolute z-score (i.e., of the 1000 total) was recorded. A null distribution of these values was formed, and the 95th percentile (i.e., *z* = ±3.911) was used as the empirical significance threshold for α = 0.05.

**2. SUPPLEMENTARY RESULTS**

**2.1 Substance-Specific Associations with SCZ2**

PRS were calculated for a revised second generation GWAS of schizophrenia (SCZ2).[^3^](#_ENREF_3) PRS calculated using CROSS[^4^](#_ENREF_4) results for SCZ demonstrated moderate to large correlations with those constructed using SCZ2 results (*r* = 0.183 at P<0.0001 to *r* = 0.538 at P≤1.0). Patterns of association for each substance did not substantially differ between CROSS-SCZ- and SCZ2-derived scores (**Table S7**). However, associations using SCZ2-derived scores tended to be significant at a wider range of P-thresholds (i.e., at the more stringent thresholds), relative to associations using CROSS-derived scores; this difference was likely the result of an increased number of SNPs being included at these more stringent thresholds (e.g., 138 SNPs in CROSS-SCZ vs. 922 SNPs in SCZ2 at P<0.0001) due to the increased power associated with the larger SCZ2 sample size.

**SUPPLEMENTARY REFERENCES**

1. Price AL, Patterson NJ, Plenge RM, Weinblatt ME, Shadick NA, Reich D. Principal components analysis corrects for stratification in genome-wide association studies. *Nature genetics* 2006; **38**(8)**:** 904-909.

2. Bierut LJ, Agrawal A, Bucholz KK, Doheny KF, Laurie C, Pugh E*, et al*. A genome-wide association study of alcohol dependence. *Proceedings of the National Academy of Sciences of the United States of America* 2010; **107**(11)**:** 5082-5087.

3. Schizophrenia Working Group of the Psychiatric Genomics Consortium. Biological insights from 108 schizophrenia-associated genetic loci. *Nature* 2014; **511**(7510)**:** 421-427.

4. Cross-Disorder Group of the Psychiatric Genomics Consortium. Identification of risk loci with shared effects on five major psychiatric disorders: a genome-wide analysis. *Lancet (London, England)* 2013; **381**(9875)**:** 1371-1379.

**Table S1. CROSS PRS and GENSUB**

| **Threshold** | ***β**** | ***SE*** | ***P*** |
| --- | --- | --- | --- |
| 0.0001 | 0.031 | 0.018 | .087 |
| 0.001 | 0.047 | 0.018 | .010 |
| 0.01 | 0.078 | 0.018 | < .001 |
| 0.05 | 0.105 | 0.019 | < .001 |
| 0.1 | 0.090 | 0.019 | < .001 |
| 0.2 | 0.099 | 0.019 | < .001 |
| 0.3 | 0.108 | 0.019 | < .001 |
| 0.4 | 0.106 | 0.019 | < .001 |
| 0.5 | 0.110 | 0.019 | < .001 |
| 1 | 0.105 | 0.019 | < .001 |

*Note.* CROSS = cross-psychiatric-disorder. GENSUB = general substance involvement liability. PRS = polygenic risk score.

**Table S2. Individual Substance Associations with ADHD PRS**

| **Substance** | **Threshold** | **OR[95%CI] for Each Involvement Level** | | | |  |
| --- | --- | --- | --- | --- | --- | --- |
|  |  | **No Symptoms** | **1-2 Symptoms** | **3-5 Symptoms** | **6-7 Symptoms** |  |
| Alcohol | 0.0001 | 1.039 [0.893-1.208] | 1.028 [0.880-1.201] | 1.099 [0.944-1.278] | 1.052 [0.900-1.229] |  |
|  | 0.001 | 0.943 [0.810-1.097] | 1.063 [0.910-1.242] | 1.036 [0.891-1.206] | 1.052 [0.900-1.230] |  |
|  | 0.01 | 0.931 [0.798-1.087] | 0.998 [0.852-1.168] | 1.062 [0.911-1.238] | 0.995 [0.850-1.165] | ^b^ |
|  | 0.05 | 0.901 [0.771-1.052] | 0.936 [0.799-1.096] | 1.064 [0.912-1.241] | 1.026 [0.875-1.203] | ^b,d^ |
|  | 0.1 | 0.890 [0.763-1.038] | 0.931 [0.796-1.090] | 1.014 [0.870-1.182] | 0.993 [0.847-1.164] | ^b^ |
|  | 0.2 | 0.914 [0.783-1.068] | 0.903 [0.770-1.058] | 1.011 [0.866-1.180] | 0.966 [0.823-1.134] |  |
|  | 0.3 | 0.898 [0.769-1.049] | 0.871 [0.743-1.021] | 0.973 [0.834-1.136] | 0.931 [0.794-1.093] |  |
|  | 0.4 | 0.919 [0.787-1.073] | 0.896 [0.765-1.050] | 0.989 [0.848-1.154] | 0.958 [0.817-1.123] |  |
|  | 0.5 | 0.907 [0.777-1.058] | 0.894 [0.763-1.046] | 0.974 [0.835-1.136] | 0.953 [0.813-1.118] |  |
|  | 1 | 0.883 [0.757-1.031] | 0.882 [0.753-1.033] | 0.967 [0.829-1.128] | 0.935 [0.797-1.096] |  |
| Cannabis | 0.0001 | 1.003 [0.906-1.110] | 1.019 [0.886-1.172] | 1.047 [0.902-1.214] | 1.006 [0.840-1.204] |  |
|  | 0.001 | 0.980 [0.885-1.085] | 1.069 [0.929-1.231] | 1.008 [0.870-1.169] | 0.950 [0.796-1.135] |  |
|  | 0.01 | 0.949 [0.856-1.051] | 1.073 [0.932-1.235] | 1.045 [0.901-1.211] | 1.059 [0.886-1.265] |  |
|  | 0.05 | 0.915 [0.826-1.015] | 1.041 [0.905-1.198] | 1.017 [0.876-1.180] | 1.055 [0.882-1.263] | ^a^ |
|  | 0.1 | 0.886 [0.799-0.982]^*†^ | 1.010 [0.877-1.162] | 0.980 [0.844-1.138] | 1.042 [0.871-1.247] | ^a^ |
|  | 0.2 | 0.874 [0.788-0.969]^*†^ | 1.013 [0.880-1.167] | 0.962 [0.828-1.118] | 0.965 [0.806-1.155] | ^a^ |
|  | 0.3 | 0.889 [0.802-0.985]^*†^ | 1.011 [0.878-1.164] | 0.936 [0.806-1.088] | 0.926 [0.773-1.110] | ^a^ |
|  | 0.4 | 0.895 [0.807-0.992]^*†^ | 1.007 [0.875-1.159] | 0.946 [0.815-1.099] | 0.948 [0.792-1.135] |  |
|  | 0.5 | 0.895 [0.808-0.992]^*†^ | 1.002 [0.870-1.154] | 0.950 [0.818-1.103] | 0.954 [0.797-1.143] |  |
|  | 1 | 0.897 [0.809-0.994]^*†^ | 0.990 [0.860-1.139] | 0.962 [0.829-1.117] | 0.969 [0.810-1.160] |  |
| Cocaine | 0.0001 | 1.039 [0.930-1.161] | 1.056 [0.843-1.322] | 0.903 [0.751-1.086] | 1.022 [0.898-1.163] |  |
|  | 0.001 | 0.995 [0.890-1.112] | 1.137 [0.905-1.429] | 1.020 [0.847-1.227] | 0.995 [0.876-1.130] |  |
|  | 0.01 | 0.966 [0.865-1.078] | 1.110 [0.887-1.388] | 1.030 [0.857-1.238] | 1.145 [1.006-1.303]^*^ | ^c^ |
|  | 0.05 | 1.041 [0.933-1.162] | 1.284 [1.026-1.607]^*^ | 1.025 [0.851-1.234] | 1.141 [1.002-1.300]^*^ |  |
|  | 0.1 | 1.009 [0.903-1.127] | 1.187 [0.949-1.487] | 1.029 [0.854-1.239] | 1.103 [0.969-1.257] |  |
|  | 0.2 | 0.989 [0.886-1.105] | 1.141 [0.913-1.426] | 0.993 [0.823-1.197] | 1.077 [0.944-1.228] |  |
|  | 0.3 | 0.989 [0.885-1.104] | 1.138 [0.912-1.419] | 1.003 [0.831-1.209] | 1.076 [0.943-1.227] |  |
|  | 0.4 | 0.992 [0.888-1.107] | 1.080 [0.866-1.347] | 0.985 [0.817-1.188] | 1.108 [0.972-1.262] |  |
|  | 0.5 | 0.979 [0.876-1.093] | 1.079 [0.865-1.347] | 0.987 [0.818-1.191] | 1.101 [0.966-1.255] |  |
|  | 1 | 0.981 [0.879-1.096] | 1.097 [0.879-1.370] | 1.004 [0.832-1.211] | 1.108 [0.973-1.263] |  |
| Nicotine | 0.0001 | 1.100 [0.916-1.320] | 1.010 [0.884-1.154] | 1.013 [0.907-1.131] | 1.138 [0.958-1.351] |  |
|  | 0.001 | 1.107 [0.921-1.332] | 1.013 [0.887-1.158] | 1.011 [0.906-1.129] | 1.037 [0.875-1.229] |  |
|  | 0.01 | 1.316 [1.094-1.582]^*†^ | 1.249 [1.092-1.429]^*†^ | 1.207 [1.079-1.349]^*†^ | 1.247 [1.051-1.480]^*†^ |  |
|  | 0.05 | 1.236 [1.027-1.488]^*†^ | 1.191 [1.041-1.364]^*†^ | 1.242 [1.108-1.391]^*†^ | 1.157 [0.973-1.375] |  |
|  | 0.1 | 1.230 [1.023-1.479]^*†^ | 1.139 [0.996-1.304] | 1.190 [1.063-1.331]^*†^ | 1.144 [0.963-1.358] |  |
|  | 0.2 | 1.279 [1.062-1.539]^*†^ | 1.153 [1.007-1.320]^*†^ | 1.187 [1.059-1.330]^*†^ | 1.232 [1.036-1.465]^*†^ |  |
|  | 0.3 | 1.254 [1.042-1.508]^*†^ | 1.163 [1.016-1.332]^*†^ | 1.173 [1.047-1.315]^*†^ | 1.232 [1.037-1.465]^*†^ |  |
|  | 0.4 | 1.253 [1.041-1.508]^*†^ | 1.151 [1.005-1.318]^*†^ | 1.174 [1.048-1.316]^*†^ | 1.263 [1.063-1.501]^*†^ |  |
|  | 0.5 | 1.298 [1.078-1.562]^*†^ | 1.161 [1.013-1.329]^*†^ | 1.180 [1.053-1.322]^*†^ | 1.315 [1.106-1.564]^*†^ |  |
|  | 1 | 1.300 [1.080-1.564]^*†^ | 1.148 [1.003-1.314]^*†^ | 1.168 [1.042-1.308]^*†^ | 1.288 [1.084-1.531]^*†^ |  |
| Opioids | 0.0001 | 1.082 [0.952-1.229] | 1.019 [0.751-1.381] | 1.053 [0.793-1.397] | 0.918 [0.762-1.106] |  |
|  | 0.001 | 1.156 [1.018-1.314]^*†^ | 0.910 [0.674-1.228] | 0.920 [0.695-1.217] | 0.905 [0.753-1.088] | ^c^ |
|  | 0.01 | 1.101 [0.971-1.250] | 0.842 [0.625-1.133] | 1.068 [0.808-1.412] | 1.102 [0.914-1.328] |  |
|  | 0.05 | 1.108 [0.976-1.259] | 0.737 [0.544-1.000] | 1.213 [0.917-1.605] | 1.094 [0.904-1.323] | ^a,d,e^ |
|  | 0.1 | 1.097 [0.966-1.246] | 0.774 [0.570-1.051] | 1.073 [0.811-1.419] | 1.033 [0.855-1.249] | ^a^ |
|  | 0.2 | 1.028 [0.905-1.168] | 0.867 [0.639-1.177] | 1.022 [0.773-1.351] | 1.009 [0.834-1.222] |  |
|  | 0.3 | 1.087 [0.956-1.235] | 0.898 [0.661-1.219] | 1.047 [0.791-1.386] | 0.956 [0.789-1.159] |  |
|  | 0.4 | 1.057 [0.931-1.201] | 0.871 [0.643-1.181] | 1.068 [0.808-1.412] | 0.943 [0.778-1.143] |  |
|  | 0.5 | 1.061 [0.934-1.206] | 0.862 [0.636-1.170] | 1.061 [0.801-1.404] | 0.960 [0.793-1.164] |  |
|  | 1 | 1.058 [0.931-1.202] | 0.867 [0.640-1.175] | 1.099 [0.831-1.452] | 0.956 [0.789-1.158] |  |

^*^ *p* < 0.05; i.e., estimate different from reference group of no/non-regular use

^†^ association remains significant at *p* < 0.05 when controlling for GENSUB ^a^ within exposed individuals, those without symptoms differ from those with 1-2 dependence symptoms

^b^ within exposed individuals, those without symptoms differ from those with 3-5 dependence symptoms

^c^ within exposed individuals, those without symptoms differ from those with 6-7 dependence symptoms

^d^ within exposed individuals, those with 1-2 dependence symptoms differ from those with 3-5 dependence symptoms

^e^ within exposed individuals, those with 1-2 dependence symptoms differ from those with 6-7 dependence symptoms

^f^ within exposed individuals, those with 3-5 dependence symptoms differ from those with 6-7 dependence symptoms

*Note.* Bolded ORs survive correction for multiple comparisons. ADHD = attention deficit hyperactivity disorder.

**Table S3. Individual Substance Associations with AUT PRS**

| **Substance** | **Threshold** | **OR[95%CI] for Each Involvement Level** | | | |  |
| --- | --- | --- | --- | --- | --- | --- |
|  |  | **No Symptoms** | **1-2 Symptoms** | **3-5 Symptoms** | **6-7 Symptoms** |  |
| Alcohol | 0.0001 | 0.953 [0.821-1.107] | 0.981 [0.840-1.147] | 0.929 [0.799-1.081] | 1.036 [0.886-1.210] |  |
|  | 0.001 | 1.109 [0.953-1.291] | 1.109 [0.948-1.296] | 1.065 [0.915-1.239] | 1.150 [0.984-1.346] |  |
|  | 0.01 | 1.084 [0.930-1.264] | 1.119 [0.957-1.309] | 1.071 [0.920-1.248] | 1.178 [1.005-1.380]^*†^ |  |
|  | 0.05 | 1.001 [0.859-1.167] | 1.050 [0.898-1.229] | 0.964 [0.827-1.124] | 1.114 [0.951-1.305] | ^f^ |
|  | 0.1 | 0.959 [0.823-1.118] | 1.036 [0.886-1.213] | 0.953 [0.818-1.111] | 1.066 [0.909-1.249] |  |
|  | 0.2 | 0.995 [0.853-1.161] | 1.028 [0.879-1.203] | 0.943 [0.809-1.099] | 1.052 [0.898-1.232] |  |
|  | 0.3 | 1.012 [0.867-1.180] | 1.062 [0.907-1.243] | 0.968 [0.831-1.129] | 1.058 [0.903-1.240] |  |
|  | 0.4 | 1.011 [0.867-1.180] | 1.074 [0.917-1.257] | 0.977 [0.838-1.139] | 1.062 [0.906-1.245] |  |
|  | 0.5 | 1.012 [0.868-1.181] | 1.094 [0.934-1.280] | 0.982 [0.842-1.145] | 1.070 [0.912-1.254] |  |
|  | 1 | 1.026 [0.879-1.197] | 1.086 [0.928-1.272] | 0.981 [0.841-1.143] | 1.066 [0.910-1.250] |  |
| Cannabis | 0.0001 | 1.020 [0.921-1.130] | 0.944 [0.820-1.087] | 0.979 [0.843-1.137] | 1.030 [0.860-1.234] |  |
|  | 0.001 | 1.051 [0.949-1.164] | 0.903 [0.784-1.040] | 1.022 [0.880-1.187] | 1.085 [0.904-1.302] | ^a^ |
|  | 0.01 | 0.916 [0.826-1.015] | 0.947 [0.822-1.090] | 1.034 [0.890-1.202] | 0.968 [0.808-1.160] |  |
|  | 0.05 | 0.976 [0.881-1.081] | 1.035 [0.899-1.192] | 0.998 [0.859-1.159] | 0.978 [0.816-1.172] |  |
|  | 0.1 | 0.983 [0.887-1.089] | 1.019 [0.885-1.173] | 1.015 [0.873-1.179] | 0.972 [0.811-1.166] |  |
|  | 0.2 | 0.975 [0.880-1.080] | 1.011 [0.878-1.163] | 0.958 [0.825-1.112] | 0.958 [0.801-1.146] |  |
|  | 0.3 | 0.967 [0.873-1.071] | 1.009 [0.877-1.161] | 0.966 [0.832-1.121] | 0.940 [0.786-1.125] |  |
|  | 0.4 | 0.961 [0.867-1.064] | 1.041 [0.905-1.198] | 0.973 [0.838-1.129] | 0.958 [0.801-1.146] |  |
|  | 0.5 | 0.962 [0.869-1.066] | 1.053 [0.915-1.212] | 0.989 [0.852-1.148] | 0.956 [0.798-1.144] |  |
|  | 1 | 0.960 [0.867-1.064] | 1.054 [0.916-1.213] | 0.984 [0.847-1.143] | 0.960 [0.802-1.150] |  |
| Cocaine | 0.0001 | 0.956 [0.855-1.070] | 0.834 [0.663-1.050] | 1.011 [0.839-1.220] | 1.060 [0.932-1.205] |  |
|  | 0.001 | 1.029 [0.920-1.150] | 0.885 [0.705-1.112] | 0.943 [0.782-1.137] | 0.993 [0.873-1.130] |  |
|  | 0.01 | 1.028 [0.920-1.149] | 0.929 [0.743-1.162] | 0.992 [0.823-1.194] | 1.012 [0.889-1.152] |  |
|  | 0.05 | 1.024 [0.917-1.145] | 0.921 [0.735-1.152] | 0.978 [0.811-1.179] | 1.042 [0.915-1.187] |  |
|  | 0.1 | 1.026 [0.918-1.147] | 0.967 [0.771-1.212] | 0.922 [0.764-1.112] | 1.057 [0.927-1.204] |  |
|  | 0.2 | 1.022 [0.914-1.142] | 1.044 [0.834-1.307] | 0.941 [0.781-1.133] | 1.028 [0.903-1.169] |  |
|  | 0.3 | 1.017 [0.910-1.136] | 1.006 [0.804-1.259] | 0.972 [0.808-1.170] | 1.021 [0.898-1.162] |  |
|  | 0.4 | 1.011 [0.905-1.130] | 1.039 [0.830-1.299] | 1.014 [0.843-1.221] | 1.033 [0.907-1.175] |  |
|  | 0.5 | 1.025 [0.917-1.145] | 1.079 [0.863-1.349] | 1.037 [0.861-1.249] | 1.034 [0.908-1.177] |  |
|  | 1 | 1.030 [0.922-1.151] | 1.085 [0.868-1.358] | 1.049 [0.870-1.263] | 1.037 [0.910-1.180] |  |
| Nicotine | 0.0001 | 1.007 [0.838-1.212] | 1.066 [0.933-1.219] | 1.082 [0.969-1.208] | 1.109 [0.935-1.316] |  |
|  | 0.001 | 1.001 [0.832-1.204] | 0.954 [0.835-1.091] | 1.014 [0.908-1.133] | 1.056 [0.890-1.254] |  |
|  | 0.01 | 1.105 [0.918-1.329] | 0.998 [0.873-1.141] | 1.060 [0.948-1.186] | 0.998 [0.841-1.185] |  |
|  | 0.05 | 1.136 [0.945-1.366] | 0.979 [0.856-1.119] | 1.029 [0.920-1.150] | 1.037 [0.873-1.231] |  |
|  | 0.1 | 0.988 [0.822-1.188] | 0.959 [0.838-1.096] | 1.009 [0.902-1.129] | 1.027 [0.864-1.220] |  |
|  | 0.2 | 0.954 [0.793-1.147] | 0.947 [0.829-1.083] | 1.009 [0.903-1.127] | 0.955 [0.805-1.133] |  |
|  | 0.3 | 0.921 [0.766-1.108] | 0.946 [0.827-1.081] | 1.008 [0.902-1.126] | 0.944 [0.796-1.121] |  |
|  | 0.4 | 0.909 [0.757-1.093] | 0.935 [0.818-1.069] | 0.987 [0.883-1.103] | 0.950 [0.801-1.128] |  |
|  | 0.5 | 0.934 [0.777-1.122] | 0.944 [0.826-1.079] | 1.006 [0.900-1.124] | 0.950 [0.800-1.128] |  |
|  | 1 | 0.932 [0.776-1.120] | 0.946 [0.827-1.081] | 1.002 [0.896-1.120] | 0.962 [0.810-1.143] |  |
| Opioids | 0.0001 | 1.007 [0.886-1.144] | 1.077 [0.794-1.462] | 1.018 [0.765-1.355] | 0.917 [0.761-1.104] |  |
|  | 0.001 | 1.041 [0.916-1.184] | 0.959 [0.703-1.309] | 0.984 [0.740-1.309] | 0.980 [0.813-1.183] |  |
|  | 0.01 | 0.979 [0.861-1.112] | 1.009 [0.742-1.371] | 0.840 [0.634-1.112] | 0.985 [0.816-1.189] |  |
|  | 0.05 | 0.908 [0.798-1.033] | 1.198 [0.879-1.633] | 0.980 [0.738-1.302] | 0.939 [0.777-1.134] |  |
|  | 0.1 | 0.900 [0.791-1.023] | 1.347 [0.987-1.839] | 0.930 [0.700-1.235] | 0.963 [0.797-1.164] | ^a^ |
|  | 0.2 | 0.883 [0.777-1.004] | 1.194 [0.880-1.621] | 0.882 [0.666-1.169] | 0.891 [0.738-1.074] |  |
|  | 0.3 | 0.899 [0.791-1.021] | 1.222 [0.901-1.657] | 0.879 [0.664-1.165] | 0.869 [0.721-1.049] |  |
|  | 0.4 | 0.917 [0.808-1.042] | 1.183 [0.872-1.606] | 0.885 [0.668-1.173] | 0.884 [0.733-1.067] |  |
|  | 0.5 | 0.929 [0.818-1.056] | 1.161 [0.856-1.576] | 0.861 [0.649-1.143] | 0.884 [0.732-1.067] |  |
|  | 1 | 0.930 [0.818-1.057] | 1.149 [0.846-1.561] | 0.844 [0.635-1.121] | 0.888 [0.735-1.073] |  |

^*^ *p* < 0.05; i.e., estimate different from reference group of no/non-regular use

^†^ association remains significant at *p* < 0.05 when controlling for GENSUB ^a^ within exposed individuals, those without symptoms differ from those with 1-2 dependence symptoms

^b^ within exposed individuals, those without symptoms differ from those with 3-5 dependence symptoms

^c^ within exposed individuals, those without symptoms differ from those with 6-7 dependence symptoms

^d^ within exposed individuals, those with 1-2 dependence symptoms differ from those with 3-5 dependence symptoms

^e^ within exposed individuals, those with 1-2 dependence symptoms differ from those with 6-7 dependence symptoms

^f^ within exposed individuals, those with 3-5 dependence symptoms differ from those with 6-7 dependence symptoms

*Note.* Bolded ORs survive correction for multiple comparisons. AUT = autism.

**Table S4. Individual Substance Associations with BIP PRS**

| **Substance** | **Threshold** | **OR[95%CI] for Each Involvement Level** | | | |  |
| --- | --- | --- | --- | --- | --- | --- |
|  |  | **No Symptoms** | **1-2 Symptoms** | **3-5 Symptoms** | **6-7 Symptoms** |  |
| Alcohol | 0.0001 | 1.026 [0.883-1.192] | 0.986 [0.844-1.153] | 0.972 [0.837-1.130] | 1.007 [0.863-1.174] |  |
|  | 0.001 | 0.911 [0.782-1.061] | 0.946 [0.808-1.107] | 0.976 [0.838-1.136] | 0.957 [0.819-1.119] |  |
|  | 0.01 | 0.973 [0.834-1.135] | 0.927 [0.791-1.085] | 1.078 [0.925-1.257] | 1.096 [0.935-1.285] | ^d,e^ |
|  | 0.05 | 0.935 [0.801-1.091] | 0.853 [0.729-0.999]^*^ | 0.980 [0.840-1.142] | 1.058 [0.903-1.239] | ^d,e^ |
|  | 0.1 | 0.985 [0.844-1.150] | 0.894 [0.763-1.048] | 0.993 [0.852-1.158] | 1.117 [0.954-1.309] | ^e,f^ |
|  | 0.2 | 0.979 [0.839-1.143] | 0.862 [0.736-1.011] | 0.981 [0.841-1.143] | 1.114 [0.951-1.305] | ^a,d,e,f^ |
|  | 0.3 | 0.992 [0.850-1.157] | 0.887 [0.757-1.039] | 0.972 [0.834-1.133] | 1.127 [0.962-1.319] | ^e,f^ |
|  | 0.4 | 0.983 [0.843-1.147] | 0.881 [0.752-1.032] | 0.965 [0.827-1.124] | 1.118 [0.955-1.308] | ^e,f^ |
|  | 0.5 | 0.984 [0.844-1.148] | 0.880 [0.751-1.031] | 0.961 [0.824-1.120] | 1.115 [0.952-1.305] | ^e,f^ |
|  | 1 | 0.969 [0.830-1.129] | 0.880 [0.751-1.030] | 0.945 [0.811-1.101] | 1.089 [0.930-1.275] | ^e,f^ |
| Cannabis | 0.0001 | 1.081 [0.977-1.195] | 1.014 [0.881-1.167] | 1.030 [0.888-1.195] | 1.022 [0.854-1.224] |  |
|  | 0.001 | 1.178 [1.062-1.306]^*†^ | 1.006 [0.873-1.160] | 1.066 [0.919-1.237] | 1.155 [0.965-1.381] | ^a^ |
|  | 0.01 | 1.158 [1.045-1.283]^*†^ | 1.059 [0.919-1.220] | 1.228 [1.057-1.427]^*^ | 1.213 [1.011-1.455]^*^ |  |
|  | 0.05 | 1.150 [1.037-1.274]^*†^ | 1.172 [1.018-1.350]^*^ | 1.244 [1.073-1.443]^*^ | 1.105 [0.924-1.321] |  |
|  | 0.1 | 1.178 [1.063-1.306]^*†^ | 1.168 [1.014-1.344]^*^ | 1.194 [1.029-1.384]^*^ | 1.119 [0.936-1.337] |  |
|  | 0.2 | 1.128 [1.018-1.250]^*†^ | 1.146 [0.996-1.319] | 1.203 [1.037-1.395]^*^ | 1.099 [0.918-1.315] |  |
|  | 0.3 | 1.122 [1.013-1.243]^*†^ | 1.119 [0.973-1.289] | 1.202 [1.037-1.394]^*^ | 1.113 [0.930-1.332] |  |
|  | 0.4 | 1.133 [1.023-1.255]^*†^ | 1.115 [0.969-1.284] | 1.179 [1.016-1.367]^*^ | 1.130 [0.944-1.352] |  |
|  | 0.5 | 1.130 [1.020-1.252]^*†^ | 1.110 [0.964-1.277] | 1.184 [1.021-1.373]^*^ | 1.134 [0.947-1.357] |  |
|  | 1 | 1.110 [1.002-1.230]^*†^ | 1.094 [0.950-1.259] | 1.158 [0.999-1.344] | 1.115 [0.931-1.335] |  |
| Cocaine | 0.0001 | 1.066 [0.954-1.192] | 0.989 [0.789-1.239] | 1.058 [0.879-1.274] | 0.968 [0.850-1.101] |  |
|  | 0.001 | 0.960 [0.860-1.073] | 1.026 [0.823-1.278] | 1.165 [0.971-1.398] | 1.061 [0.933-1.207] |  |
|  | 0.01 | 0.976 [0.874-1.090] | 1.080 [0.865-1.348] | 1.281 [1.062-1.545]^*^ | 1.176 [1.031-1.341]^*^ | ^b,c^ |
|  | 0.05 | 0.986 [0.882-1.102] | 1.190 [0.952-1.486] | 1.231 [1.023-1.481]^*^ | 1.202 [1.058-1.367]^*^ | ^b,c^ |
|  | 0.1 | 1.002 [0.897-1.120] | 1.204 [0.966-1.501] | 1.172 [0.975-1.408] | 1.177 [1.036-1.338]^*^ | ^c^ |
|  | 0.2 | 0.973 [0.871-1.088] | 1.239 [0.993-1.546] | 1.202 [0.999-1.447] | 1.205 [1.058-1.371]^*^ | ^a,b,c^ |
|  | 0.3 | 0.975 [0.872-1.090] | 1.213 [0.973-1.511] | 1.150 [0.956-1.384] | 1.204 [1.058-1.369]^*^ | ^c^ |
|  | 0.4 | 0.981 [0.878-1.097] | 1.242 [0.995-1.549] | 1.181 [0.981-1.422] | 1.227 [1.079-1.396]^*^ | ^a,c^ |
|  | 0.5 | 0.984 [0.880-1.100] | 1.224 [0.981-1.526] | 1.186 [0.985-1.428] | 1.245 [1.094-1.416]^*^ | ^c^ |
|  | 1 | 0.977 [0.874-1.092] | 1.244 [0.997-1.551] | 1.161 [0.964-1.399] | 1.227 [1.078-1.396]^*^ | ^a,c^ |
| Nicotine | 0.0001 | 0.988 [0.824-1.184] | 1.015 [0.889-1.159] | 1.058 [0.949-1.179] | 0.960 [0.811-1.137] |  |
|  | 0.001 | 1.108 [0.924-1.329] | 1.027 [0.899-1.174] | 1.028 [0.921-1.148] | 0.986 [0.832-1.169] |  |
|  | 0.01 | 0.952 [0.793-1.143] | 0.992 [0.868-1.135] | 1.061 [0.948-1.187] | 1.047 [0.882-1.244] |  |
|  | 0.05 | 0.998 [0.830-1.201] | 1.044 [0.913-1.194] | 1.102 [0.986-1.231] | 1.117 [0.942-1.324] |  |
|  | 0.1 | 1.007 [0.838-1.210] | 1.035 [0.906-1.184] | 1.103 [0.987-1.232] | 1.089 [0.919-1.291] |  |
|  | 0.2 | 0.928 [0.772-1.116] | 1.012 [0.885-1.158] | 1.066 [0.954-1.191] | 1.066 [0.899-1.264] |  |
|  | 0.3 | 0.908 [0.756-1.091] | 1.009 [0.883-1.154] | 1.054 [0.943-1.177] | 1.069 [0.902-1.267] |  |
|  | 0.4 | 0.942 [0.784-1.132] | 1.030 [0.901-1.177] | 1.078 [0.965-1.205] | 1.103 [0.930-1.307] |  |
|  | 0.5 | 0.942 [0.784-1.132] | 1.042 [0.912-1.192] | 1.089 [0.974-1.216] | 1.105 [0.932-1.310] |  |
|  | 1 | 0.929 [0.773-1.117] | 1.018 [0.890-1.164] | 1.067 [0.955-1.192] | 1.080 [0.911-1.280] |  |
| Opioids | 0.0001 | 0.964 [0.849-1.094] | 1.091 [0.806-1.478] | 0.918 [0.693-1.217] | 1.026 [0.851-1.237] |  |
|  | 0.001 | 0.886 [0.780-1.006] | 1.387 [1.037-1.854]^*^ | 1.031 [0.779-1.363] | 1.013 [0.840-1.221] | ^a^ |
|  | 0.01 | 0.990 [0.871-1.125] | 1.516 [1.117-2.056]^*^ | 1.039 [0.780-1.383] | 1.148 [0.948-1.390] | ^a^ |
|  | 0.05 | 1.009 [0.889-1.146] | 1.394 [1.039-1.871]^*^ | 1.005 [0.760-1.329] | 1.155 [0.960-1.391] | ^a^ |
|  | 0.1 | 1.011 [0.891-1.147] | 1.484 [1.106-1.991]^*^ | 0.982 [0.742-1.300] | 1.114 [0.925-1.341] | ^a,d^ |
|  | 0.2 | 0.992 [0.874-1.126] | 1.479 [1.097-1.995]^*^ | 0.968 [0.730-1.283] | 1.117 [0.926-1.346] | ^a,d^ |
|  | 0.3 | 0.989 [0.871-1.122] | 1.497 [1.113-2.014]^*^ | 0.998 [0.753-1.324] | 1.078 [0.894-1.299] | ^a,d^ |
|  | 0.4 | 0.988 [0.871-1.122] | 1.476 [1.095-1.990]^*^ | 1.043 [0.786-1.383] | 1.100 [0.912-1.327] | ^a^ |
|  | 0.5 | 0.985 [0.868-1.119] | 1.481 [1.101-1.994]^*^ | 1.057 [0.797-1.403] | 1.099 [0.911-1.326] | ^a^ |
|  | 1 | 0.963 [0.847-1.093] | 1.452 [1.078-1.956]^*^ | 1.035 [0.779-1.375] | 1.068 [0.885-1.288] | ^a^ |

^*^ *p* < 0.05; i.e., estimate different from reference group of no/non-regular use

^†^ association remains significant at *p* < 0.05 when controlling for GENSUB ^a^ within exposed individuals, those without symptoms differ from those with 1-2 dependence symptoms

^b^ within exposed individuals, those without symptoms differ from those with 3-5 dependence symptoms

^c^ within exposed individuals, those without symptoms differ from those with 6-7 dependence symptoms

^d^ within exposed individuals, those with 1-2 dependence symptoms differ from those with 3-5 dependence symptoms

^e^ within exposed individuals, those with 1-2 dependence symptoms differ from those with 6-7 dependence symptoms

^f^ within exposed individuals, those with 3-5 dependence symptoms differ from those with 6-7 dependence symptoms

*Note.* Bolded ORs survive correction for multiple comparisons. BIP = bipolar disorder.

**Table S5. Individual Substance Associations with MDD PRS**

| **Substance** | **Threshold** | **OR[95%CI] for Each Involvement Level** | | | |  |
| --- | --- | --- | --- | --- | --- | --- |
|  |  | **No Symptoms** | **1-2 Symptoms** | **3-5 Symptoms** | **6-7 Symptoms** |  |
| Alcohol | 0.0001 | 1.123 [0.964-1.308] | 1.130 [0.967-1.322] | 1.149 [0.987-1.337] | 1.230 [1.052-1.438]^*†^ |  |
|  | 0.001 | 1.064 [0.914-1.238] | 0.982 [0.840-1.148] | 1.066 [0.916-1.241] | 1.195 [1.022-1.398]^*^ | ^e^ |
|  | 0.01 | 1.058 [0.906-1.235] | 0.946 [0.806-1.109] | 1.093 [0.936-1.275] | 1.076 [0.917-1.262] | ^d^ |
|  | 0.05 | 1.085 [0.925-1.273] | 0.945 [0.802-1.113] | 1.147 [0.979-1.344] | 1.157 [0.983-1.361] | ^a,d,e^ |
|  | 0.1 | 1.048 [0.892-1.231] | 0.938 [0.795-1.107] | 1.172 [0.999-1.375] | 1.149 [0.975-1.353] | ^d,e^ |
|  | 0.2 | 1.009 [0.859-1.186] | 0.896 [0.759-1.058] | 1.188 [1.011-1.395]^*^ | 1.214 [1.029-1.431]^*^ | ^b,c,d,e^ |
|  | 0.3 | 1.020 [0.868-1.200] | 0.905 [0.765-1.069] | 1.176 [1.001-1.382]^*^ | 1.230 [1.042-1.451]^*^ | ^b,c,d,e^ |
|  | 0.4 | 1.037 [0.882-1.219] | 0.895 [0.758-1.058] | 1.179 [1.003-1.385]^*^ | 1.264 [1.071-1.491]^*^ | ^a,b,c,d,e^ |
|  | 0.5 | 1.046 [0.889-1.230] | 0.924 [0.782-1.093] | 1.203 [1.024-1.414]^*^ | 1.290 [1.093-1.523]^*^ | ^b,c,d,e^ |
|  | 1 | 1.054 [0.896-1.239] | 0.925 [0.781-1.094] | 1.220 [1.037-1.434]^*^ | 1.285 [1.089-1.517]^*^ | ^b,c,d,e^ |
| Cannabis | 0.0001 | 1.053 [0.951-1.166] | 1.009 [0.877-1.160] | 0.976 [0.842-1.132] | 1.154 [0.964-1.381] |  |
|  | 0.001 | 1.143 [1.032-1.266]^*^ | 1.167 [1.014-1.344]^*^ | 1.054 [0.908-1.223] | 1.258 [1.049-1.508]^*^ |  |
|  | 0.01 | 1.121 [1.010-1.243]^*^ | 1.095 [0.949-1.264] | 1.012 [0.869-1.179] | 1.221 [1.014-1.469]^*^ |  |
|  | 0.05 | 1.161 [1.044-1.290]^*^ | 1.069 [0.924-1.237] | 1.149 [0.986-1.340] | 1.041 [0.865-1.252] |  |
|  | 0.1 | 1.193 [1.072-1.327]^*^ | 1.121 [0.969-1.299] | 1.171 [1.004-1.366]^*^ | 1.128 [0.936-1.359] |  |
|  | 0.2 | **1.240 [1.114-1.380]^*^** | 1.151 [0.994-1.334] | 1.210 [1.036-1.413]^*†^ | 1.225 [1.016-1.479]^*†^ |  |
|  | 0.3 | 1.236 [1.110-1.377]^*^ | 1.139 [0.982-1.320] | 1.206 [1.032-1.408]^*^ | 1.250 [1.036-1.508]^*^ |  |
|  | 0.4 | **1.254 [1.126-1.398]^*^** | 1.142 [0.985-1.325] | 1.223 [1.047-1.430]^*^ | 1.255 [1.040-1.515]^*†^ |  |
|  | 0.5 | **1.267 [1.137-1.412]^*^** | 1.157 [0.997-1.342] | 1.226 [1.048-1.434]^*^ | 1.262 [1.045-1.524]^*†^ |  |
|  | 1 | **1.263 [1.133-1.407]^*^** | 1.155 [0.995-1.341] | 1.227 [1.049-1.435]^*^ | 1.258 [1.042-1.519]^*†^ |  |
| Cocaine | 0.0001 | 1.017 [0.911-1.136] | 0.986 [0.786-1.235] | 1.305 [1.083-1.573]^*^ | 1.028 [0.904-1.169] | ^b,f^ |
|  | 0.001 | 1.110 [0.994-1.240] | 0.994 [0.793-1.247] | 1.223 [1.014-1.476]^*^ | 1.124 [0.988-1.280] |  |
|  | 0.01 | 1.084 [0.969-1.213] | 0.919 [0.732-1.154] | 1.127 [0.931-1.363] | 1.166 [1.021-1.331]^*^ |  |
|  | 0.05 | 1.116 [0.995-1.252] | 0.947 [0.751-1.195] | 1.144 [0.945-1.385] | 1.280 [1.119-1.464]^*†^ | ^e^ |
|  | 0.1 | 1.134 [1.010-1.273]^*†^ | 1.074 [0.853-1.354] | 1.209 [0.998-1.464] | 1.290 [1.127-1.476]^*†^ |  |
|  | 0.2 | 1.136 [1.011-1.278]^*^ | 1.111 [0.879-1.405] | 1.293 [1.064-1.571]^*†^ | **1.394 [1.215-1.599]^*†^** | ^c^ |
|  | 0.3 | 1.107 [0.985-1.245] | 1.063 [0.840-1.346] | 1.238 [1.020-1.503]^*^ | **1.381 [1.205-1.584]^*†^** | ^c,e^ |
|  | 0.4 | 1.096 [0.973-1.233] | 1.032 [0.814-1.309] | 1.190 [0.979-1.446] | **1.391 [1.212-1.596]^*†^** | ^c,e^ |
|  | 0.5 | 1.108 [0.984-1.248] | 1.054 [0.830-1.338] | 1.163 [0.957-1.415] | **1.401 [1.221-1.608]^*†^** | ^c,e^ |
|  | 1 | 1.123 [0.997-1.265] | 1.048 [0.826-1.329] | 1.178 [0.969-1.432] | **1.398 [1.218-1.603]^*†^** | ^c,e^ |
| Nicotine | 0.0001 | 1.027 [0.855-1.234] | 0.931 [0.814-1.064] | 0.981 [0.878-1.096] | 0.896 [0.756-1.063] |  |
|  | 0.001 | 1.121 [0.933-1.347] | 1.064 [0.930-1.216] | 1.104 [0.988-1.234] | 1.122 [0.946-1.332] |  |
|  | 0.01 | 0.992 [0.823-1.196] | 0.964 [0.841-1.105] | 1.082 [0.966-1.212] | 1.047 [0.879-1.246] | ^d^ |
|  | 0.05 | 1.087 [0.899-1.313] | 0.956 [0.832-1.098] | 1.070 [0.954-1.199] | 1.136 [0.953-1.354] | ^d^ |
|  | 0.1 | 1.137 [0.940-1.376] | 0.964 [0.838-1.108] | 1.085 [0.967-1.217] | 1.184 [0.993-1.413] | ^d,e^ |
|  | 0.2 | 1.194 [0.985-1.447] | 0.955 [0.831-1.099] | 1.122 [1.000-1.259] | 1.227 [1.027-1.466]^*^ | ^a,d,e^ |
|  | 0.3 | 1.166 [0.962-1.414] | 0.952 [0.827-1.096] | 1.117 [0.995-1.254] | 1.264 [1.058-1.510]^*^ | ^a,d,e^ |
|  | 0.4 | 1.200 [0.989-1.457] | 0.983 [0.854-1.132] | 1.140 [1.016-1.280]^*^ | 1.288 [1.078-1.540]^*^ | ^a,d,e^ |
|  | 0.5 | 1.198 [0.987-1.455] | 1.016 [0.882-1.171] | 1.151 [1.024-1.292]^*^ | 1.293 [1.081-1.546]^*^ | ^d,e^ |
|  | 1 | 1.220 [1.005-1.482]^*^ | 1.004 [0.872-1.157] | 1.153 [1.027-1.295]^*^ | 1.290 [1.079-1.542]^*^ | ^a,d,e^ |
| Opioids | 0.0001 | 1.116 [0.982-1.267] | 1.013 [0.748-1.373] | 1.111 [0.838-1.474] | 1.039 [0.862-1.250] |  |
|  | 0.001 | 1.071 [0.942-1.217] | 1.066 [0.788-1.443] | 0.998 [0.750-1.328] | 1.179 [0.977-1.423] |  |
|  | 0.01 | 0.938 [0.823-1.068] | 0.999 [0.734-1.360] | 1.088 [0.814-1.454] | 1.004 [0.828-1.218] |  |
|  | 0.05 | 1.014 [0.890-1.156] | 1.056 [0.777-1.436] | 1.231 [0.924-1.640] | 1.022 [0.842-1.240] |  |
|  | 0.1 | 1.039 [0.911-1.184] | 1.025 [0.753-1.394] | 1.249 [0.936-1.668] | 1.130 [0.930-1.372] |  |
|  | 0.2 | 1.091 [0.955-1.245] | 1.152 [0.841-1.578] | 1.329 [0.990-1.784] | 1.186 [0.975-1.443] |  |
|  | 0.3 | 1.056 [0.925-1.205] | 1.085 [0.793-1.486] | 1.332 [0.994-1.785] | 1.194 [0.982-1.452] |  |
|  | 0.4 | 1.033 [0.905-1.180] | 1.107 [0.807-1.518] | 1.309 [0.976-1.755] | 1.218 [1.001-1.481]^*^ |  |
|  | 0.5 | 1.047 [0.916-1.196] | 1.108 [0.807-1.522] | 1.324 [0.988-1.775] | 1.219 [1.002-1.483]^*^ |  |
|  | 1 | 1.045 [0.915-1.194] | 1.117 [0.816-1.530] | 1.295 [0.967-1.735] | 1.191 [0.979-1.448] |  |

^*^ *p* < 0.05; i.e., estimate different from reference group of no/non-regular use

^†^ association remains significant at *p* < 0.05 when controlling for GENSUB ^a^ within exposed individuals, those without symptoms differ from those with 1-2 dependence symptoms

^b^ within exposed individuals, those without symptoms differ from those with 3-5 dependence symptoms

^c^ within exposed individuals, those without symptoms differ from those with 6-7 dependence symptoms

^d^ within exposed individuals, those with 1-2 dependence symptoms differ from those with 3-5 dependence symptoms

^e^ within exposed individuals, those with 1-2 dependence symptoms differ from those with 6-7 dependence symptoms

^f^ within exposed individuals, those with 3-5 dependence symptoms differ from those with 6-7 dependence symptoms

*Note.* Bolded ORs survive correction for multiple comparisons. MDD = major depressive disorder.

**Table S6. Individual Substance Associations with SCZ PRS**

| **Substance** | **Threshold** | **OR[95%CI] for Each Involvement Level** | | | |  |
| --- | --- | --- | --- | --- | --- | --- |
|  |  | **No Symptoms** | **1-2 Symptoms** | **3-5 Symptoms** | **6-7 Symptoms** |  |
| Alcohol | 0.0001 | 0.933 [0.801-1.086] | 1.020 [0.873-1.192] | 1.024 [0.880-1.192] | 1.022 [0.873-1.196] |  |
|  | 0.001 | 1.000 [0.858-1.165] | 0.942 [0.805-1.101] | 0.977 [0.839-1.137] | 1.025 [0.877-1.198] |  |
|  | 0.01 | 0.915 [0.785-1.066] | 0.915 [0.781-1.072] | 0.944 [0.811-1.100] | 1.025 [0.876-1.198] | ^b,c,d,e^ |
|  | 0.05 | 0.842 [0.722-0.982]^*†^ | 0.872 [0.744-1.023] | 0.996 [0.854-1.160] | 1.073 [0.917-1.256] | ^b,c,e,f^ |
|  | 0.1 | 0.878 [0.754-1.024] | 0.912 [0.778-1.069] | 1.016 [0.871-1.184] | 1.157 [0.988-1.354] | ^b,c,e,f^ |
|  | 0.2 | 0.927 [0.795-1.081] | 0.999 [0.851-1.173] | 1.086 [0.930-1.268] | 1.295 [1.104-1.519]^*^ | ^b,c,e,f^ |
|  | 0.3 | 0.909 [0.779-1.059] | 0.992 [0.846-1.164] | 1.062 [0.910-1.239] | 1.265 [1.079-1.483]^*^ | ^b,c,e,f^ |
|  | 0.4 | 0.891 [0.764-1.039] | 0.980 [0.835-1.151] | 1.053 [0.902-1.229] | 1.248 [1.064-1.463]^*^ | ^b,c,e,f^ |
|  | 0.5 | 0.905 [0.776-1.055] | 1.000 [0.852-1.174] | 1.067 [0.914-1.246] | 1.269 [1.082-1.488]^*^ | ^b,c,e,f^ |
|  | 1 | 0.899 [0.771-1.050] | 1.007 [0.858-1.183] | 1.070 [0.917-1.250] | 1.265 [1.078-1.484]^*^ | ^b,c,e,f^ |
| Cannabis | 0.0001 | 1.104 [0.997-1.223] | 1.027 [0.892-1.182] | 1.038 [0.895-1.204] | 1.049 [0.877-1.254] |  |
|  | 0.001 | 1.164 [1.050-1.289]^*†^ | 1.040 [0.903-1.198] | 1.076 [0.929-1.246] | 1.196 [1.004-1.424]^*^ |  |
|  | 0.01 | 1.208 [1.089-1.339]^*†^ | 1.091 [0.946-1.258] | 1.169 [1.007-1.358]^*^ | 1.335 [1.115-1.598]^*^ | ^e^ |
|  | 0.05 | **1.235 [1.113-1.371]^*†^** | 1.166 [1.009-1.347]^*^ | 1.160 [0.996-1.351] | **1.466 [1.221-1.759]^*^** | ^e,f^ |
|  | 0.1 | 1.218 [1.097-1.353]^*^ | 1.177 [1.017-1.362]^*^ | 1.236 [1.061-1.441]^*^ | **1.506 [1.252-1.811]^*^** | ^c,e^ |
|  | 0.2 | **1.255 [1.130-1.395]^*^** | 1.186 [1.024-1.374]^*^ | 1.305 [1.118-1.523]^*^ | **1.561 [1.295-1.882]^*^** | ^c,e^ |
|  | 0.3 | **1.250 [1.126-1.389]^*^** | 1.171 [1.011-1.356]^*^ | 1.319 [1.130-1.539]^*^ | **1.522 [1.261-1.835]^*^** | ^c,e^ |
|  | 0.4 | **1.261 [1.136-1.401]^*†^** | 1.205 [1.040-1.395]^*^ | 1.348 [1.155-1.574]^*^ | **1.502 [1.245-1.812]^*^** | ^e^ |
|  | 0.5 | **1.271 [1.144-1.413]^*†^** | 1.209 [1.044-1.401]^*^ | 1.347 [1.154-1.572]^*^ | **1.496 [1.241-1.805]^*^** | ^e^ |
|  | 1 | **1.283 [1.155-1.427]^*†^** | 1.230 [1.061-1.425]^*^ | **1.366 [1.170-1.595]^*^** | **1.505 [1.247-1.816]^*^** |  |
| Cocaine | 0.0001 | 1.028 [0.920-1.150] | 0.890 [0.712-1.113] | 0.951 [0.789-1.146] | 0.986 [0.867-1.122] |  |
|  | 0.001 | 0.991 [0.885-1.110] | 1.028 [0.816-1.294] | 0.935 [0.779-1.122] | 1.061 [0.936-1.202] |  |
|  | 0.01 | 1.049 [0.937-1.175] | 1.161 [0.921-1.462] | 0.961 [0.799-1.157] | 1.223 [1.074-1.392]^*^ | ^f^ |
|  | 0.05 | 1.040 [0.928-1.167] | 1.187 [0.941-1.499] | 1.104 [0.913-1.334] | **1.355 [1.187-1.546]^*^** | ^c^ |
|  | 0.1 | 1.016 [0.905-1.141] | 1.258 [0.993-1.594] | 1.080 [0.891-1.310] | **1.474 [1.289-1.686]^*†^** | ^c,f^ |
|  | 0.2 | 1.024 [0.911-1.150] | 1.279 [1.011-1.619]^*^ | 1.108 [0.912-1.346] | **1.473 [1.286-1.688]^*^** | ^c,f^ |
|  | 0.3 | 0.999 [0.889-1.122] | 1.314 [1.039-1.664]^*^ | 1.081 [0.889-1.314] | **1.467 [1.280-1.681]^*^** | ^a,c,f^ |
|  | 0.4 | 1.014 [0.903-1.139] | 1.316 [1.041-1.663]^*^ | 1.126 [0.926-1.368] | **1.500 [1.308-1.720]^*†^** | ^a,c,f^ |
|  | 0.5 | 1.004 [0.894-1.128] | 1.318 [1.043-1.665]^*^ | 1.120 [0.922-1.360] | **1.498 [1.307-1.717]^*†^** | ^a,c,f^ |
|  | 1 | 1.013 [0.901-1.138] | 1.308 [1.036-1.651]^*^ | 1.122 [0.923-1.364] | **1.515 [1.321-1.737]^*†^** | ^a,c,f^ |
| Nicotine | 0.0001 | 1.119 [0.931-1.346] | 1.151 [1.007-1.315]^*^ | 1.103 [0.988-1.233] | 1.197 [1.009-1.421]^*^ |  |
|  | 0.001 | 1.003 [0.832-1.208] | 1.054 [0.923-1.204] | 1.070 [0.960-1.193] | 1.147 [0.969-1.358] |  |
|  | 0.01 | 1.077 [0.895-1.298] | 1.068 [0.934-1.222] | 1.083 [0.970-1.209] | 1.139 [0.961-1.351] |  |
|  | 0.05 | 1.082 [0.898-1.304] | 1.108 [0.967-1.269] | 1.123 [1.004-1.255]^*^ | 1.141 [0.961-1.354] |  |
|  | 0.1 | 1.140 [0.946-1.375] | 1.124 [0.981-1.288] | 1.133 [1.013-1.267]^*^ | 1.209 [1.017-1.436]^*^ |  |
|  | 0.2 | 1.179 [0.978-1.421] | 1.148 [1.001-1.317]^*^ | 1.149 [1.026-1.286]^*^ | 1.292 [1.086-1.537]^*^ |  |
|  | 0.3 | 1.208 [1.002-1.456]^*^ | 1.161 [1.012-1.332]^*^ | 1.166 [1.041-1.306]^*^ | 1.311 [1.102-1.560]^*^ |  |
|  | 0.4 | 1.224 [1.016-1.475]^*^ | 1.173 [1.022-1.345]^*^ | 1.179 [1.053-1.320]^*^ | 1.305 [1.097-1.553]^*^ |  |
|  | 0.5 | 1.230 [1.021-1.482]^*^ | 1.173 [1.022-1.345]^*^ | 1.181 [1.054-1.322]^*^ | 1.299 [1.092-1.545]^*^ |  |
|  | 1 | 1.249 [1.036-1.505]^*^ | 1.196 [1.042-1.373]^*^ | 1.186 [1.059-1.329]^*^ | 1.308 [1.099-1.557]^*^ |  |
| Opioids | 0.0001 | 0.952 [0.837-1.082] | 1.356 [0.998-1.842] | 1.023 [0.770-1.360] | 1.047 [0.868-1.263] | ^a^ |
|  | 0.001 | 1.061 [0.935-1.204] | 0.966 [0.718-1.299] | 0.971 [0.738-1.278] | 1.137 [0.949-1.362] |  |
|  | 0.01 | 1.135 [0.999-1.290] | 1.258 [0.932-1.697] | 0.913 [0.690-1.208] | 1.173 [0.975-1.412] |  |
|  | 0.05 | 1.207 [1.060-1.374]^*^ | 1.404 [1.039-1.897]^*^ | 1.045 [0.786-1.389] | 1.204 [0.998-1.452] |  |
|  | 0.1 | 1.191 [1.045-1.358]^*^ | 1.355 [1.002-1.831]^*^ | 1.164 [0.874-1.551] | 1.248 [1.033-1.506]^*^ |  |
|  | 0.2 | **1.239 [1.086-1.413]^*^** | 1.320 [0.975-1.787] | 1.148 [0.857-1.537] | 1.208 [0.997-1.463] |  |
|  | 0.3 | 1.193 [1.045-1.361]^*^ | 1.295 [0.954-1.758] | 1.118 [0.835-1.497] | 1.209 [0.998-1.465] |  |
|  | 0.4 | 1.213 [1.063-1.384]^*^ | 1.318 [0.972-1.787] | 1.107 [0.827-1.484] | 1.168 [0.963-1.416] |  |
|  | 0.5 | 1.207 [1.058-1.376]^*^ | 1.330 [0.982-1.802] | 1.107 [0.827-1.481] | 1.149 [0.948-1.393] |  |
|  | 1 | 1.229 [1.078-1.402]^*^ | 1.312 [0.968-1.778] | 1.131 [0.844-1.515] | 1.135 [0.935-1.377] |  |

^*^ *p* < 0.05; i.e., estimate different from reference group of no/non-regular use

^†^ association remains significant at *p* < 0.05 when controlling for GENSUB ^a^ within exposed individuals, those without symptoms differ from those with 1-2 dependence symptoms

^b^ within exposed individuals, those without symptoms differ from those with 3-5 dependence symptoms

^c^ within exposed individuals, those without symptoms differ from those with 6-7 dependence symptoms

^d^ within exposed individuals, those with 1-2 dependence symptoms differ from those with 3-5 dependence symptoms

^e^ within exposed individuals, those with 1-2 dependence symptoms differ from those with 6-7 dependence symptoms

^f^ within exposed individuals, those with 3-5 dependence symptoms differ from those with 6-7 dependence symptoms

*Note.* Bolded ORs survive correction for multiple comparisons. SCZ = schizophrenia.

**Table S7. Individual Substance Associations with SCZ2 PRS**

| **Substance** | **Threshold** | **OR[95%CI] for Each Involvement Level** | | | |  |
| --- | --- | --- | --- | --- | --- | --- |
|  |  | **No Symptoms** | **1-2 Symptoms** | **3-5 Symptoms** | **6-7 Symptoms** |  |
| Alcohol | 0.0001 | 0.906 [0.775-1.060] | 1.009 [0.860-1.184] | 1.058 [0.906-1.236] | 1.260 [1.073-1.479]^*^ | ^b,c,e,f^ |
|  | 0.001 | 0.872 [0.746-1.020] | 0.945 [0.806-1.108] | 1.032 [0.883-1.205] | 1.218 [1.035-1.432]^*^ | ^b,c,e,f^ |
|  | 0.01 | 0.873 [0.744-1.023] | 0.951 [0.808-1.118] | 1.014 [0.866-1.187] | 1.211 [1.029-1.425]^*^ | ^b,c,e,f^ |
|  | 0.05 | 0.872 [0.744-1.022] | 0.917 [0.779-1.079] | 1.011 [0.863-1.184] | 1.269 [1.078-1.494]^*^ | ^b,c,e,f^ |
|  | 0.1 | 0.852 [0.726-0.999]^*^ | 0.881 [0.748-1.039] | 0.993 [0.848-1.164] | 1.267 [1.076-1.493]^*^ | ^b,c,e,f^ |
|  | 0.2 | 0.856 [0.729-1.004] | 0.907 [0.769-1.070] | 0.993 [0.846-1.164] | 1.262 [1.071-1.488]^*^ | ^b,c,e,f^ |
|  | 0.3 | 0.853 [0.727-1.002] | 0.918 [0.778-1.083] | 1.001 [0.853-1.175] | 1.285 [1.090-1.516]^*^ | ^b,c,e,f^ |
|  | 0.4 | 0.857 [0.730-1.008] | 0.923 [0.782-1.090] | 1.016 [0.865-1.193] | 1.295 [1.097-1.528]^*^ | ^b,c,e,f^ |
|  | 0.5 | 0.852 [0.724-1.001] | 0.920 [0.779-1.087] | 1.017 [0.866-1.194] | 1.287 [1.090-1.519]^*^ | ^b,c,e,f^ |
|  | 1 | 0.845 [0.719-0.993]^*†^ | 0.919 [0.778-1.085] | 1.004 [0.855-1.180] | 1.280 [1.085-1.511]^*^ | ^b,c,e,f^ |
| Cannabis | 0.0001 | 1.191 [1.073-1.322]^*^ | 1.160 [1.005-1.338]^*^ | 1.208 [1.039-1.404]^*^ | 1.350 [1.126-1.617]^*^ |  |
|  | 0.001 | 1.213 [1.093-1.346]^*^ | 1.100 [0.952-1.271] | 1.244 [1.066-1.450]^*^ | 1.389 [1.153-1.673]^*^ | ^e^ |
|  | 0.01 | 1.202 [1.081-1.336]^*^ | 1.102 [0.953-1.274] | 1.313 [1.128-1.529]^*^ | 1.369 [1.142-1.642]^*^ | ^d,e^ |
|  | 0.05 | 1.164 [1.046-1.295]^*^ | 1.044 [0.901-1.211] | 1.361 [1.166-1.589]^*^ | 1.372 [1.139-1.652]^*^ | ^b,d,e^ |
|  | 0.1 | 1.185 [1.064-1.319]^*^ | 1.091 [0.940-1.267] | **1.388 [1.188-1.621]^*^** | 1.340 [1.111-1.615]^*^ | ^b,d,e^ |
|  | 0.2 | 1.196 [1.074-1.333]^*^ | 1.124 [0.967-1.306] | **1.430 [1.221-1.674]^*^** | 1.401 [1.159-1.693]^*^ | ^b,d,e^ |
|  | 0.3 | 1.220 [1.094-1.360]^*^ | 1.132 [0.974-1.317] | **1.463 [1.249-1.714]^*^** | 1.418 [1.172-1.716]^*^ | ^b,d,e^ |
|  | 0.4 | 1.229 [1.101-1.371]^*^ | 1.142 [0.981-1.329] | **1.463 [1.248-1.715]^*^** | 1.425 [1.177-1.726]^*^ | ^b,d,e^ |
|  | 0.5 | 1.228 [1.101-1.370]^*^ | 1.152 [0.989-1.340] | **1.455 [1.241-1.705]^*^** | 1.402 [1.158-1.697]^*^ | ^b,d^ |
|  | 1 | 1.231 [1.103-1.373]^*^ | 1.151 [0.989-1.340] | **1.459 [1.245-1.710]^*^** | 1.405 [1.161-1.702]^*^ | ^b,d^ |
| Cocaine | 0.0001 | 0.961 [0.859-1.076] | 0.962 [0.763-1.212] | 1.148 [0.952-1.385] | 1.215 [1.066-1.385]^*^ | ^c^ |
|  | 0.001 | 1.034 [0.923-1.158] | 1.042 [0.825-1.318] | 1.144 [0.943-1.387] | 1.237 [1.081-1.414]^*^ | ^c^ |
|  | 0.01 | 1.073 [0.957-1.203] | 1.260 [0.999-1.590] | 1.189 [0.986-1.435] | 1.255 [1.100-1.431]^*^ | ^c^ |
|  | 0.05 | 1.103 [0.981-1.240] | 1.205 [0.951-1.526] | 1.154 [0.952-1.399] | **1.364 [1.193-1.559]^*^** | ^c^ |
|  | 0.1 | 1.133 [1.007-1.275]^*^ | 1.168 [0.920-1.483] | 1.124 [0.926-1.364] | **1.351 [1.181-1.546]^*^** | ^c^ |
|  | 0.2 | 1.164 [1.034-1.312]^*^ | 1.188 [0.933-1.512] | 1.222 [1.004-1.487]^*^ | **1.403 [1.224-1.608]^*^** | ^c^ |
|  | 0.3 | 1.189 [1.055-1.340]^*^ | 1.170 [0.918-1.492] | 1.245 [1.022-1.517]^*^ | **1.421 [1.239-1.630]^*^** | ^c^ |
|  | 0.4 | 1.185 [1.051-1.336]^*^ | 1.187 [0.931-1.513] | 1.252 [1.027-1.526]^*^ | **1.415 [1.233-1.623]^*^** | ^c^ |
|  | 0.5 | 1.176 [1.043-1.325]^*^ | 1.178 [0.924-1.502] | 1.240 [1.018-1.511]^*^ | **1.403 [1.223-1.609]^*^** | ^c^ |
|  | 1 | 1.154 [1.024-1.301]^*^ | 1.184 [0.928-1.511] | 1.243 [1.020-1.515]^*^ | **1.400 [1.221-1.606]^*^** | ^c^ |
| Nicotine | 0.0001 | 1.326 [1.099-1.599]^*†^ | 1.164 [1.015-1.334]^*^ | 1.191 [1.063-1.334]^*^ | **1.566 [1.314-1.866]^*†^** | ^e,f^ |
|  | 0.001 | 1.198 [0.993-1.447] | 1.119 [0.976-1.284] | 1.112 [0.992-1.246] | **1.461 [1.222-1.747]^*^** | ^e,f^ |
|  | 0.01 | 1.230 [1.017-1.487]^*^ | 1.178 [1.026-1.351]^*^ | 1.105 [0.986-1.238] | 1.414 [1.187-1.685]^*^ | ^e,f^ |
|  | 0.05 | 1.260 [1.040-1.527]^*^ | 1.158 [1.008-1.332]^*^ | 1.128 [1.006-1.264]^*^ | 1.379 [1.155-1.646]^*^ | ^f^ |
|  | 0.1 | 1.222 [1.007-1.481]^*^ | 1.137 [0.989-1.308] | 1.128 [1.006-1.266]^*^ | 1.409 [1.180-1.683]^*^ | ^e,f^ |
|  | 0.2 | 1.260 [1.038-1.530]^*^ | 1.153 [1.001-1.327]^*^ | 1.167 [1.039-1.310]^*^ | 1.388 [1.161-1.661]^*^ | ^e,f^ |
|  | 0.3 | 1.265 [1.041-1.538]^*^ | 1.189 [1.032-1.370]^*^ | 1.193 [1.061-1.341]^*^ | **1.437 [1.200-1.720]^*^** | ^e,f^ |
|  | 0.4 | 1.295 [1.065-1.574]^*^ | 1.183 [1.026-1.364]^*^ | 1.178 [1.048-1.324]^*^ | **1.434 [1.197-1.718]^*^** | ^e,f^ |
|  | 0.5 | 1.280 [1.053-1.557]^*^ | 1.189 [1.031-1.371]^*^ | 1.183 [1.052-1.330]^*^ | **1.441 [1.203-1.726]^*^** | ^e,f^ |
|  | 1 | 1.285 [1.057-1.563]^*^ | 1.191 [1.033-1.374]^*^ | 1.185 [1.054-1.333]^*^ | **1.450 [1.210-1.736]^*^** | ^e,f^ |
| Opioids | 0.0001 | 1.119 [0.984-1.273] | 1.196 [0.885-1.615] | 1.158 [0.875-1.534] | 1.320 [1.091-1.596]^*^ |  |
|  | 0.001 | 1.141 [0.999-1.303] | 1.254 [0.916-1.717] | 0.985 [0.740-1.312] | 1.293 [1.064-1.571]^*^ |  |
|  | 0.01 | 1.183 [1.039-1.347]^*^ | 1.578 [1.164-2.139]^*†^ | 1.108 [0.834-1.471] | 1.278 [1.058-1.544]^*^ |  |
|  | 0.05 | 1.138 [0.997-1.299] | 1.377 [1.011-1.876]^*^ | 1.178 [0.880-1.578] | 1.305 [1.077-1.582]^*^ |  |
|  | 0.1 | 1.154 [1.010-1.317]^*^ | 1.398 [1.026-1.906]^*^ | 1.112 [0.828-1.494] | 1.267 [1.045-1.536]^*^ |  |
|  | 0.2 | 1.158 [1.013-1.325]^*^ | 1.407 [1.028-1.927]^*^ | 1.127 [0.836-1.521] | 1.283 [1.056-1.559]^*^ |  |
|  | 0.3 | 1.178 [1.030-1.349]^*^ | 1.458 [1.063-2.000]^*^ | 1.133 [0.839-1.532] | 1.302 [1.071-1.583]^*^ |  |
|  | 0.4 | 1.181 [1.032-1.352]^*^ | 1.398 [1.018-1.920]^*^ | 1.155 [0.854-1.562] | 1.303 [1.071-1.585]^*^ |  |
|  | 0.5 | 1.180 [1.031-1.350]^*^ | 1.401 [1.022-1.919]^*^ | 1.143 [0.846-1.546] | 1.294 [1.065-1.574]^*^ |  |
|  | 1 | 1.179 [1.030-1.350]^*^ | 1.418 [1.034-1.945]^*^ | 1.160 [0.858-1.569] | 1.283 [1.055-1.560]^*^ |  |

^*^ *p* < 0.05; i.e., estimate different from reference group of no/non-regular use

^†^ association remains significant at *p* < 0.05 when controlling for GENSUB ^a^ within exposed individuals, those without symptoms differ from those with 1-2 dependence symptoms

^b^ within exposed individuals, those without symptoms differ from those with 3-5 dependence symptoms

^c^ within exposed individuals, those without symptoms differ from those with 6-7 dependence symptoms

^d^ within exposed individuals, those with 1-2 dependence symptoms differ from those with 3-5 dependence symptoms

^e^ within exposed individuals, those with 1-2 dependence symptoms differ from those with 6-7 dependence symptoms

^f^ within exposed individuals, those with 3-5 dependence symptoms differ from those with 6-7 dependence symptoms

*Note.* Bolded ORs survive correction for multiple comparisons. SCZ = schizophrenia.

**Table S8. Individual Substance Associations with MDD PRS, Controlling for MDD Diagnosis**

| **Substance** | **Threshold** | **OR[95%CI] for Each Involvement Level** | | | |  |
| --- | --- | --- | --- | --- | --- | --- |
|  |  | **No Symptoms** | **1-2 Symptoms** | **3-5 Symptoms** | **6-7 Symptoms** |  |
| Alcohol | 0.0001 | 1.120 [0.961-1.305] | 1.128 [0.964-1.319] | 1.154 [0.991-1.343] | 1.237 [1.057-1.447]^*^ |  |
|  | 0.001 | 1.060 [0.910-1.235] | 0.981 [0.839-1.147] | 1.075 [0.923-1.252] | 1.204 [1.029-1.410]^*^ | ^e^ |
|  | 0.01 | 1.070 [0.916-1.250] | 0.954 [0.814-1.120] | 1.102 [0.944-1.287] | 1.084 [0.923-1.272] | ^d^ |
|  | 0.05 | 1.098 [0.936-1.289] | 0.954 [0.809-1.125] | 1.153 [0.984-1.352] | 1.165 [0.989-1.371] | ^a,d,e^ |
|  | 0.1 | 1.058 [0.900-1.244] | 0.947 [0.802-1.117] | 1.175 [1.001-1.380]^*^ | 1.155 [0.980-1.362] | ^d,e^ |
|  | 0.2 | 1.019 [0.867-1.198] | 0.904 [0.766-1.068] | 1.192 [1.015-1.400]^*^ | 1.220 [1.034-1.439]^*^ | ^b,c,d,e^ |
|  | 0.3 | 1.032 [0.877-1.214] | 0.914 [0.773-1.080] | 1.180 [1.004-1.388]^*^ | 1.235 [1.047-1.458]^*^ | ^b,c,d,e^ |
|  | 0.4 | 1.050 [0.893-1.235] | 0.906 [0.766-1.071] | 1.183 [1.007-1.391]^*^ | 1.271 [1.077-1.501]^*^ | ^a,c,d,e^ |
|  | 0.5 | 1.058 [0.900-1.245] | 0.934 [0.790-1.105] | 1.207 [1.026-1.419]^*^ | 1.296 [1.097-1.530]^*^ | ^b,c,d,e^ |
|  | 1 | 1.067 [0.907-1.256] | 0.935 [0.790-1.107] | 1.225 [1.041-1.440]^*^ | 1.292 [1.094-1.526]^*^ | ^b,c,d,e^ |
| Cannabis | 0.0001 | 1.057 [0.954-1.170] | 1.012 [0.879-1.164] | 0.979 [0.844-1.135] | 1.157 [0.966-1.385] |  |
|  | 0.001 | 1.150 [1.038-1.275]^*^ | 1.172 [1.018-1.349]^*^ | 1.056 [0.910-1.226] | 1.259 [1.050-1.510]^*^ |  |
|  | 0.01 | 1.124 [1.013-1.247]^*^ | 1.097 [0.950-1.266] | 1.010 [0.867-1.177] | 1.219 [1.012-1.468]^*^ |  |
|  | 0.05 | 1.161 [1.044-1.290]^*^ | 1.067 [0.922-1.235] | 1.146 [0.983-1.337] | 1.038 [0.862-1.250] |  |
|  | 0.1 | 1.194 [1.073-1.328]^*^ | 1.120 [0.967-1.297] | 1.167 [1.000-1.361] | 1.124 [0.933-1.354] |  |
|  | 0.2 | **1.242 [1.116-1.383]^*^** | 1.150 [0.992-1.332] | 1.206 [1.032-1.409]^*†^ | 1.220 [1.011-1.472]^*†^ |  |
|  | 0.3 | 1.237 [1.110-1.378]^*^ | 1.137 [0.981-1.318] | 1.201 [1.028-1.403]^*^ | 1.244 [1.031-1.502]^*^ |  |
|  | 0.4 | **1.256 [1.127-1.399]^*^** | 1.141 [0.983-1.323] | 1.218 [1.042-1.424]^*^ | 1.249 [1.034-1.508]^*^ |  |
|  | 0.5 | **1.268 [1.138-1.413]^*^** | 1.155 [0.995-1.341] | 1.221 [1.043-1.428]^*^ | 1.255 [1.039-1.517]^*†^ |  |
|  | 1 | **1.264 [1.134-1.409]^*^** | 1.154 [0.994-1.339] | 1.223 [1.046-1.430]^*^ | 1.253 [1.038-1.514]^*^ |  |
| Cocaine | 0.0001 | 1.018 [0.912-1.138] | 0.993 [0.791-1.246] | 1.305 [1.082-1.573]^*^ | 1.028 [0.904-1.169] | ^b,f^ |
|  | 0.001 | 1.114 [0.997-1.245] | 1.013 [0.807-1.272] | 1.224 [1.015-1.478]^*^ | 1.126 [0.989-1.282] |  |
|  | 0.01 | 1.083 [0.968-1.212]^*†^ | 0.924 [0.735-1.162] | 1.127 [0.932-1.364] | 1.168 [1.022-1.333]^*^ |  |
|  | 0.05 | 1.114 [0.993-1.250]^*†^ | 0.951 [0.753-1.200] | 1.144 [0.945-1.385] | 1.280 [1.119-1.464]^*†^ | ^e^ |
|  | 0.1 | 1.130 [1.006-1.269] | 1.093 [0.866-1.378] | 1.208 [0.997-1.463] | 1.289 [1.127-1.476]^*^ |  |
|  | 0.2 | 1.134 [1.008-1.275] | 1.128 [0.891-1.428] | 1.292 [1.064-1.570]^*†^ | **1.394 [1.215-1.600]^*†^** | ^c^ |
|  | 0.3 | 1.104 [0.981-1.242] | 1.075 [0.849-1.361] | 1.237 [1.019-1.502]^*^ | **1.382 [1.206-1.585]^*†^** | ^c^ |
|  | 0.4 | 1.092 [0.970-1.229] | 1.044 [0.822-1.325] | 1.189 [0.979-1.445] | **1.392 [1.213-1.596]^*†^** | ^c,e^ |
|  | 0.5 | 1.104 [0.980-1.244] | 1.065 [0.838-1.353] | 1.163 [0.956-1.415] | **1.402 [1.222-1.609]^*†^** | ^c,e^ |
|  | 1 | 1.120 [0.994-1.262] | 1.055 [0.831-1.340] | 1.179 [0.969-1.433] | **1.399 [1.219-1.604]^*†^** | ^c,e^ |
| Nicotine | 0.0001 | 1.029 [0.856-1.236] | 0.932 [0.815-1.065] | 0.984 [0.881-1.099] | 0.903 [0.761-1.071] |  |
|  | 0.001 | 1.121 [0.933-1.348] | 1.066 [0.932-1.219] | 1.109 [0.992-1.240] | 1.126 [0.948-1.337] |  |
|  | 0.01 | 0.997 [0.827-1.202] | 0.969 [0.845-1.111] | 1.086 [0.969-1.217] | 1.044 [0.876-1.245] | ^d^ |
|  | 0.05 | 1.091 [0.903-1.318] | 0.962 [0.837-1.105] | 1.071 [0.955-1.200] | 1.135 [0.952-1.354] |  |
|  | 0.1 | 1.141 [0.943-1.381] | 0.972 [0.845-1.118] | 1.086 [0.968-1.218] | 1.182 [0.990-1.411] | ^e^ |
|  | 0.2 | 1.198 [0.989-1.452] | 0.963 [0.837-1.108] | 1.123 [1.000-1.261]^*^ | 1.222 [1.021-1.461]^*^ | ^a,d,e^ |
|  | 0.3 | 1.171 [0.966-1.419] | 0.959 [0.833-1.104] | 1.117 [0.995-1.255] | 1.258 [1.052-1.505]^*^ | ^a,d,e^ |
|  | 0.4 | 1.206 [0.994-1.464] | 0.992 [0.861-1.142] | 1.141 [1.016-1.281]^*^ | 1.283 [1.072-1.536]^*^ | ^a,d,e^ |
|  | 0.5 | 1.204 [0.992-1.462] | 1.025 [0.890-1.181] | 1.151 [1.025-1.293]^*^ | 1.286 [1.074-1.539]^*^ | ^d,e^ |
|  | 1 | 1.227 [1.010-1.489]^*^ | 1.013 [0.879-1.167] | 1.155 [1.028-1.297]^*^ | 1.285 [1.074-1.538]^*^ | ^d,e^ |
| Opioids | 0.0001 | 1.119 [0.985-1.271] | 1.012 [0.747-1.370] | 1.112 [0.839-1.475] | 1.040 [0.863-1.252] |  |
|  | 0.001 | 1.075 [0.946-1.222] | 1.063 [0.785-1.438] | 0.998 [0.750-1.328] | 1.181 [0.978-1.425] |  |
|  | 0.01 | 0.937 [0.822-1.067] | 0.994 [0.729-1.356] | 1.087 [0.813-1.453] | 1.004 [0.828-1.218] |  |
|  | 0.05 | 1.013 [0.889-1.155] | 1.055 [0.775-1.435] | 1.229 [0.923-1.638] | 1.021 [0.842-1.239] |  |
|  | 0.1 | 1.041 [0.913-1.187] | 1.023 [0.751-1.393] | 1.248 [0.935-1.667] | 1.129 [0.930-1.371] |  |
|  | 0.2 | 1.092 [0.956-1.247] | 1.150 [0.839-1.576] | 1.327 [0.989-1.782] | 1.185 [0.974-1.441] |  |
|  | 0.3 | 1.055 [0.924-1.205] | 1.082 [0.790-1.483] | 1.330 [0.993-1.782] | 1.193 [0.981-1.451] |  |
|  | 0.4 | 1.033 [0.904-1.180] | 1.103 [0.804-1.515] | 1.307 [0.974-1.753] | 1.217 [1.000-1.480]^*^ |  |
|  | 0.5 | 1.045 [0.914-1.195] | 1.105 [0.804-1.518] | 1.322 [0.986-1.773] | 1.218 [1.001-1.482]^*^ |  |
|  | 1 | 1.044 [0.914-1.193] | 1.115 [0.813-1.528] | 1.294 [0.966-1.733] | 1.190 [0.978-1.447] |  |

^*^ *p* < 0.05; i.e., estimate different from reference group of no/non-regular use

^†^ association remains significant at *p* < 0.05 when controlling for GENSUB ^a^ within exposed individuals, those without symptoms differ from those with 1-2 dependence symptoms

^b^ within exposed individuals, those without symptoms differ from those with 3-5 dependence symptoms

^c^ within exposed individuals, those without symptoms differ from those with 6-7 dependence symptoms

^d^ within exposed individuals, those with 1-2 dependence symptoms differ from those with 3-5 dependence symptoms

^e^ within exposed individuals, those with 1-2 dependence symptoms differ from those with 6-7 dependence symptoms

^f^ within exposed individuals, those with 3-5 dependence symptoms differ from those with 6-7 dependence symptoms

*Note.* Bolded ORs survive correction for multiple comparisons. MDD = major depressive disorder.

**Figure S1. PRS Distributions at P<0.3**

**
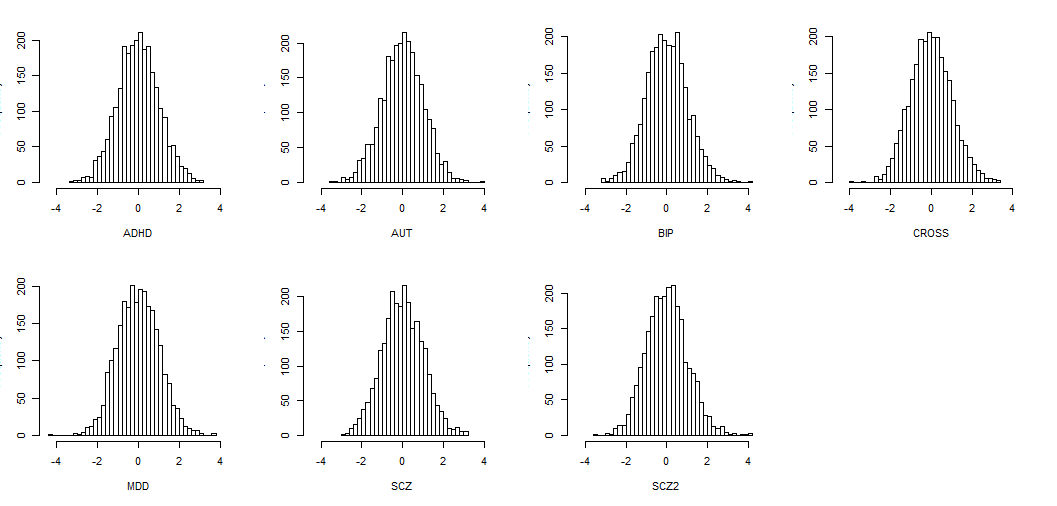
**

PRS = Polygenic risk scores. ADHD = attention deficit hyperactivity disorder. AUT = autism. BIP = bipolar disorder. MDD = major depressive disorder. SCZ = schizophrenia.

**Figure S2. Factor Loadings for the Confirmatory One-Factor Model of GENSUB**


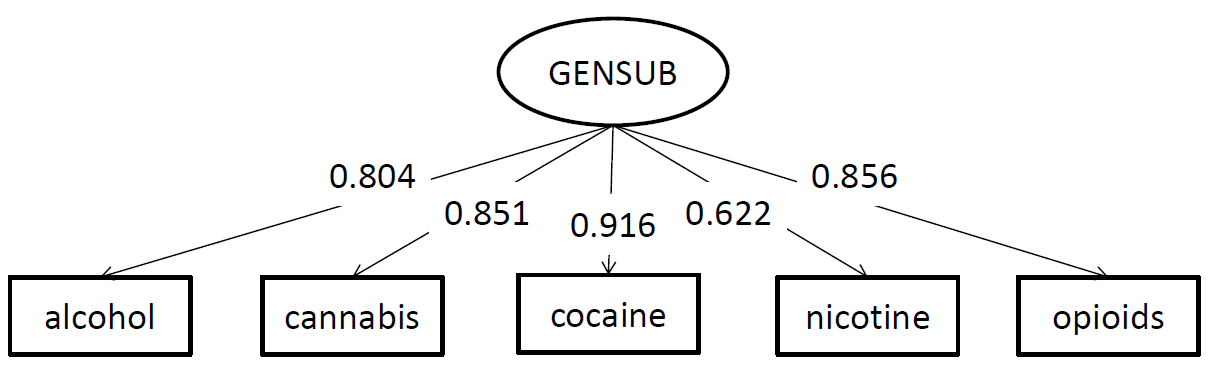


**Figure S3. Comparison of CROSS SCZ to SCZ2 PRS Results**


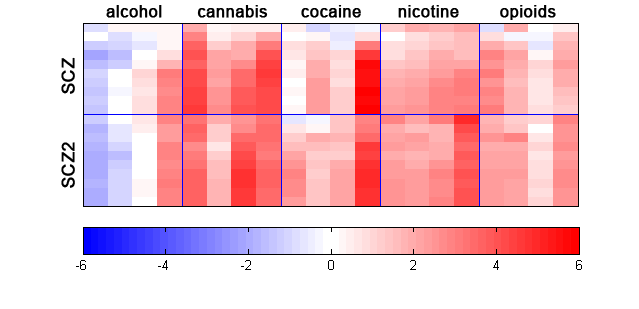


Associations between individual substance involvement and polygenic risk scores for SCZ derived from the cross-disorder meta-analysis and second-generation PGC GWAS. Within each grid space, P-thresholds (0.0001, 0.001, 0.01, 0.05, 0.1, 0.2, 0.3, 0.4, 0.5, and 1.0) are represented vertically in ascending order. Levels of involvement (i.e., no/non-regular use, use without endorsement of any dependence symptoms, 1-2 dependence symptoms, 3-5 dependence symptoms, and 6-7 dependence symptoms) are represented horizontally in ascending order. Colors represent z-scores for each association test, with no lifetime or nonregular use as the reference group. Post-hoc Wald tests comparing all levels of substance involvement with one another are reported in **Tables S6 and S7**. SCZ = schizophrenia.
